# The reach of road salt into vernal pools and the response of amphibians

**DOI:** 10.1371/journal.pone.0329680

**Published:** 2025-10-23

**Authors:** Noah D. Charney, Charles S. Eiseman, Ethan B. Plunkett, Sydne Record, Paige S. Warren

**Affiliations:** 1 Department of Wildlife, Fisheries, and Conservation Biology, University of Maine, Orono, Maine, United States of America; 2 Maine Agricultural and Forest Experiment Station, Orono, Maine, United States of America; 3 Organismic and Evolutionary Biology Program, University of Massachusetts, Amherst, Massachusetts, United States of America; 4 Independent Researcher, Northfield, Massachusetts, United States of America; 5 Department of Environmental Conservation, University of Massachusetts, Amherst, Massachusetts, United States of America; Charles University: Univerzita Karlova, CZECHIA

## Abstract

Deicing salts are causing freshwater wetlands to become increasingly saline near roadways, with cascading impacts on local ecosystems. Understanding the full reach of salt into surrounding landscapes can inform the decisions made every winter about roadway management. We measured conductivity and surveyed for vernal-pool obligate species at 541 wetlands identified as potential vernal pools in western Massachusetts, USA, estimating that the salt effect zone extends as far as 167 m to 251 m from roadways. For the smallest wetlands with perimeters under 100 m, the salt effect zone extends to between 81 and 128 m. The mean conductivity of wetlands beyond 251 m was 91 μS/cm (SD = 109 μS/cm), whereas mean conductivity was 168 μS/cm (SD = 180 μS/cm) between 167 m and 251 m, and 274 μS/cm (SD = 340 μS/cm) at wetlands within 167 m of roads. Occupancy and N-Mixture models found that the threefold higher conductivities in average wetlands within the salt effect zone would cause 14% (SE = 5%) lower predicted rates of site occupancy for spotted salamander (*Ambystoma maculatum*), 15% (SE = 5%) lower occupancy for wood frogs (*Lithobates sylvatica*), 29% (SE = 11%) lower population sizes for spotted salamanders and 19% (SE = 14%) lower population sizes for wood frogs, although the wood frog abundance model did not meet the threshold for statistical significance. Compared to average wetlands, the mean conductivity was lower in wetlands with marbled salamanders (*Ambystoma opacum*) and fairy shrimp (*Eubranchipus* sp.) and approximately the same for Jefferson salamanders (*Ambystoma jeffersonianum* and associated unisexual *Ambystoma*), but data for these species were insufficient for formal occupancy modeling. We estimate that 78% of all vernal pools in Massachusetts fall within the road salt effect zone, underscoring the importance of integrating decision-making surrounding roadways and conservation.

## Introduction

The ecological impacts of roads include local habitat alterations, barriers to wildlife movement, roadkill mortality, chemical runoff, noise pollution, facilitation of nonnative species spread, and other effects [1–7]. Although roads themselves only cover approximately 1% of the United States, Forman [8] estimated that the area ecologically impacted by roads amounts to approximately 20% of the country.

Identifying the distances to which effects of roads extend will aid managers in evaluating the environmental impact of new road construction, identifying zones of high ecological integrity, designing structural features to limit runoff, establishing areas of reduced roadway chemical application, and assisting with other mitigation efforts [2]. In regions with snowy winters, runoff of deicing salts is of particular concern for both the potential to alter local ecology as well as the consequences for human health [9–14], and is part of a broader trend in global salinization of freshwater wetlands [15]. Road salt runoff has been shown to impact nearby vegetation [16–18], aquatic microbial communities [19], aquatic invertebrates [20], and amphibian communities [21]. In a mesocosm experiment, Jones et al [22] found that salt-induced declines in zooplankton led to increased phytoplankton growth.

Road salt effects are better documented for amphibians than for other taxa, as researchers work to identify the underlying causes of global amphibian declines [23,24]. Laboratory studies have shown that increased salt concentrations increase the frequency of physical abnormalities for spotted salamanders (*Ambystoma maculatum*)*,* green frogs (*Lithobates clamitans*) and wood frogs (*L. sylvaticus*) [25,26], decrease survival and time to metamorphosis in *L. sylvaticus* [25,27] and induce morphological changes in *L. sylvaticus* [28]*.* In a transplant field experiment, where *A. maculatum* eggs were transferred from woodland pools (>50 m from a road) to roadside pools (within 2 m of a road), Turtle [29] observed reduced survival of embryos in roadside pools that had higher salt concentrations compared to woodland pools that had lower salt concentrations. With field manipulations, Karraker et al. [30] found that embryonic and larval survival decreased at intermediate (500 μs/cm) and high (3000 μs/cm) concentrations for *A. maculatum* and at high concentrations for *L. sylvaticus*. These studies demonstrate that deicing salt can substantially impact local amphibian populations near roads, although few studies have aimed to characterize how far these effects extend at the landscape level. In this study, we aimed to characterize the distance from roads at which runoff alters aquatic chemistry of northeastern vernal pools and to estimate the potential magnitude of influence on wetland-dependent species.

## Methods

### Study system

Ephemeral wetlands known as “vernal pools” in eastern forests are host to a unique assemblage of specialist animals. Vernal pools are typically small, isolated from other wetlands, seasonally dry, detritus-driven, and contain few fish or other long-lived predatory species [31]. Species usually considered obligate vernal pool breeders include wood frog (*Lithobates sylvaticus*), spotted salamander (*Ambystoma maculatum*), marbled salamander (*A. opacum*), members of the Jefferson/unisexual salamander complex (henceforth “Jefferson salamander,” *A. jeffersonianum* and unisexual *Ambystoma* associates), fairy shrimp (*Eubranchipus* spp.), and others. Because of these unique communities, vernal pools are the focus of much public and private conservation effort [32,33].

**Fig 1:**
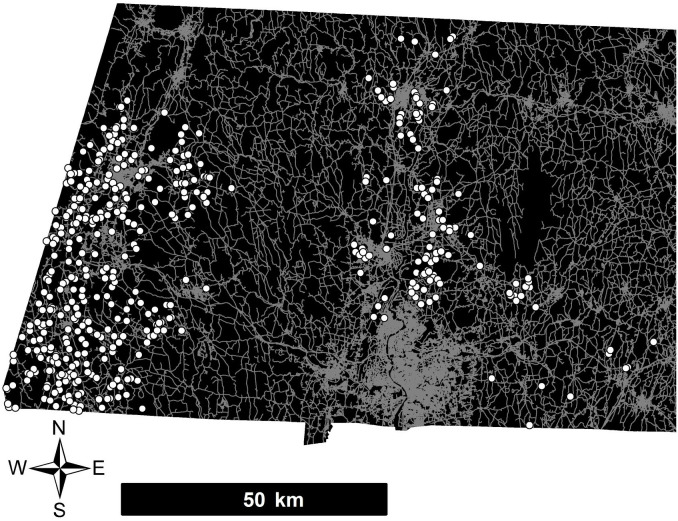
Map of surveyed ponds (white dots). The black area is western Massachusetts and gray lines are roads.

Our study region of western Massachusetts contains approximately one million people and 20,000 km of roads within an area of 9,500 km^2^ (Fig 1). Dominant land covers in 2005 as mapped by MassGIS in this region are forest (75%), agriculture (8%) and residential (7%; www.mass.gov). The mean percent impervious surface measured in 1-km cells at our sample locations is 4.1% (sd = 5%, min = 0%, max = 55%) (Elvidge et al. 2007). Mean annual snowfall averaged across weather stations in our study area is approximately 1.5 m/year (www.ncdc.noaa.gov).

### Field data collection

We identified 541 wetlands in the Connecticut River Valley and the Berkshires regions using the Massachusetts potential vernal pool GIS data layer [34](PVP; www.massgis.gov) and through incidental encounters while walking to sites (n = 34). We performed diurnal visual surveys between April 2 and May 17 in 2008, 2009 and 2010. To minimize bias due to spatial and temporal autocorrelation, for most pond visits, we assigned survey dates such that ponds visited within a local area within a few days of each other spanned the full range of local and regional habitat fragmentation indices described for these ponds by Compton et al. [35]. We visited each wetland once, measuring water conductivity using OAKTON Instruments PTTestr35 meters (Vernon Hills, Illinois, U.S.A.). This instrument reported “total dissolved solutes,” which we converted to conductivity by dividing by 0.71, following manufacturer guidelines which are based on the physical principle that dissolved ions in water facilitate the flow of electrical current through water [36]. We recorded coordinates of each sampled wetland in the field with Garmin 76CSx handheld GPS devices. We walked the entire perimeter of each wetland inspecting for signs of wood frogs, spotted salamanders, Jefferson salamanders, marbled salamanders, and fairy shrimp. We recorded our paces in the field to measure perimeter and then estimated wetland area by combining this with a shape complexity index derived from a sketch of the shoreline. Acceptable signs of presence included presence of adults, larvae, egg masses or spermatophores. Because it is difficult to identify spermatophores to species, we assigned all spermatophore-only detections to spotted salamanders, as they are much more common in our region. For each amphibian species observed, we counted the number of egg masses visible from our perimeter surveys. Facing into the uplands at the four cardinal directions from the pond’s edge, we visually estimated percent tree canopy cover within 30 m of the pond edge. To model detection error, in 2009 we revisited 51 ponds sampled in 2008. No permits were necessary for this work, as it did not require handling organisms, we obeyed all posted signs, and Massachusetts law generally allows public access to private or public land that is not legally posted (Massachusetts G.L. c. 266 § 120: Trespass).

### Spatial data

In ArcMap 10.0, we measured the distance from every pond to the nearest roads included on the Massachusetts Department of Transportation Roads data layer, which includes all roads that the state maintains and encompasses all public and most private thoroughfares (www.mass.gov). We used the USGS bedrock lithology layer to identify the bedrock type occurring at each pond. We then classified this as a binary variable indicating whether the natural ground waters in contact with each bedrock type tended to be high-solute/high-pH, as described by Robinson & Kapo [37]. At each pond, we extracted soil nutrient availability, mapped as “SQ1” by the Harmonized World Soil Database [38]. While these data are mapped only at a coarse 1-km grid size, it was the best available soil data covering our region. Prior to regression analyses, to obtain a more normal distribution in the response variable, we log-transformed water conductivity and to obtain a more linear relationship between response and predictor, we log-transformed the distance to nearest road.

### Road effect zone

To determine the road effect zone, we estimated the distance past which further increasing distance from road no longer causes a detectable decline in water conductivity. To accomplish this, we first performed a linear mixed-effects regression on all 541 wetlands using conductivity as the response variable, distance from road, local forest canopy cover, elevation, soil category, and bedrock category as fixed effects and region as a random effect using the lme4 library in R [39]. We also examined models *post hoc* containing additional covariates of wetland perimeter, wetland area, day of year, and the National Land Cover Database 2008 land cover type occurring at the wetland coordinates, but the model we chose *a priori* had the lowest AICc out of all possible parameter combinations examined using the dredge function in the MuMIn package of R [40]. We also examined the full model with non-transformed distance from road, but this model had an overall worse fit than the log-transformed response (ΔAIC = 12). After fitting the initial model with all wetlands, we iteratively fit the model excluding any wetlands within a given threshold distance from roads. We adjusted this threshold from 1 m (including all 541 wetlands) up to the distance at which there were too few wetlands to fit the model (which occurred at 1056 m, when there were only 20 wetlands left). We then defined the end of the potential road effect zone as the distance at which the estimated effect of distance from road was no longer different from zero in the models.

### Species response

To determine the impact of water conductivity on vernal pool species, we used the unmarked package in R [41] to conduct occupancy analyses for the species with sufficient detections – spotted salamanders and wood frogs. Because vernal-pool breeding amphibians are long lived, have high site fidelity, and may skip breeding years [42–44], we interpret occupancy as population presence at a patch centered around the breeding pond, not as merely breeding attempts. Thus, occupied patches may not have egg masses in the pond in any given year, so we treat visits across years as multiple observations in a “single-season” modeling framework [45]. We included as occupancy covariates conductivity, distance from road, elevation, perimeter length, and forest cover, with day of year, and canopy cover as detection covariates. We also examined occupancy models that included conductivity in the detection model to account for any potential differences in detectability related to water clarity resulting from underlying water quality. Based on a prior scale analysis that included these data [46], we used 1650 m radius circular buffers for spotted salamanders and 1150 m radius buffers for wood frogs, applied to the 2011 National Land Cover Database percent tree canopy layer [47]. We tested the goodness of fit of occupancy models using parametric bootstrapping with the unmarked::parboot() function [48]. To investigate potential effects of conductivity on abundance of the spotted salamanders and wood frogs, we then used the same set of predictor variables in the same occupancy framework with an N-mixture model [49] with egg mass count as the response and a 150 m radius buffer for forest canopy cover. We also used AIC to examine whether a quadratic term of conductivity would improve the abundance models to account for unimodal response. Otherwise, all detection and state variables remained the same between N-mixture and occupancy models. We evaluated overdispersion in models with zero-inflated Poisson and negative binomial error distributions using the chat() function in the nmixgof R package [50].$\sqrt{\hat{\text{c}}}$ prior to reporting and prior to calculating p-values and goodness of fit was checked with parametric bootstrapping using parboot(). All independent variables were scaled by subtracting by the mean and dividing by the standard deviation prior to modeling. To determine whether each variable should be log-transformed prior to fitting the full model, we fit single-predictor simple linear regressions to each species and summed delta-AICs across the two species for models fit with log-transformed versus raw predictor values. Based on the resultant AIC values, we log transformed conductivity, perimeter, and canopy cover only. Due to missing covariates, primarily perimeter, only 485 of the wetlands were used for the occupancy models.

## Results

The distance from wetlands to the nearest road ranged from 1 m to 1839 m (mean = 253, sd = 294). Water conductivity ranged from 3 μS/cm to 2690 μS/cm (mean = 200 μS/cm, SD = 277 μS/cm). The mean pond perimeter was 177 m (SD = 132 m), the mean pond surface area was 2,100 m^2^ (SD = 3,600), and the estimated percent forest canopy within 30 m of ponds ranged from 0% to 100% (mean = 62%, sd = 30%). There were 283 ponds occurring on high-pH/high-solute rock types, primarily carbonate rocks, while most of the remaining 258 ponds occurred primarily on variety of metamorphic and igneous bedrock types. Wetlands occurred on soil nutrient availability categories 1 (n = 86), 2 (n = 97), 3 (n = 344), and 4 (n = 10), with four wetlands occurring on soils classified as water bodies.

**Fig 2:**
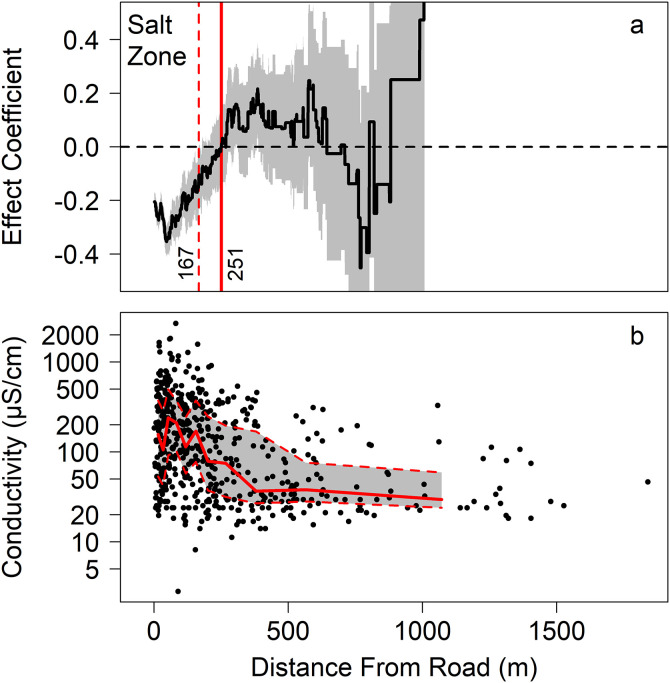
(a) For wetlands further than a given distance from the nearest road (x-axis) the estimated effect (y-axis) of distance from road (log-transformed) on conductivity (log-transformed) in a linear mixed model. Vertical lines indicate the distance beyond which moving further from roads no longer results in reduced conductivity. The mean estimates are represented by solid lines with standard errors represented by gray shading and dotted lines. **(b)** The raw conductivity versus the distance from road for all 541 wetlands used in the model, sampled in 2008, 2009, and 2010 in western Massachusetts, USA. Solid line represents median over a 50-sample moving window, whereas dotted lines represent the upper and lower quartiles.

Distance from road had a significant negative effect on water conductivity (p << 0.001; Table 1). This effect disappeared from our model entirely at 251 m from the nearest road, with standard errors overlapping zero beginning at 167 m (Fig 2). The choice to log-transform the conductivity response did not change the mean estimate of the salt effect zone, but when conductivity was not log-transformed, the uncertainty was higher as a result of poorer-fitting models. Perimeters were obtained for 502 of the wetlands, and when considering only the smallest 172 wetlands with perimeters under 100 m, the estimated road effect zone ended at between 81 m and 126 m. Using only the 330 wetlands with perimeters over 100 m, the road effect zone ended between 167 and 275 m. If we include only the 387 wetlands where vernal pool indicator species were detected, the road effect zone ended between 167 and 231 m. If we include only the 320 wetlands that contained indicator species other than spotted salamanders, which are known to be more adaptable to different wetland types [51], the road effect zone ended between 110 and 220 m.

**Table 1:** Parameter estimates from logistic regression of the log of water conductivity, measured in μS/cm at 541 wetlands in western Massachusetts, USA sampled in spring of 2008, 2009, and 2010, with variables scaled prior to modeling using the original mean and standard deviations listed.

Type	Effect	Mean	SD	Units	Estimate	SE	df	t	*p*
Fixed	Intercept				4.41	0.56	1.05	7.42	0.08
Fixed	log(Dist. from Road)	4.9	1.2	log(m)	−0.24	0.04	483	−5.91	<< 0.001
Fixed	Forest Canopy	62	30	%	−0.28	0.04	483	−6.88	<<0.001
Fixed	Elevation	289	159	m	−0.59	0.07	471	−8.15	<< 0.001
Fixed	Soil SQ1 Category 2				0.17	0.13	483	1.32	0.2
Fixed	Soil SQ1 Category 3				−0.32	0.11	484	−2.86	0.004
Fixed	Soil SQ1 Category 4				−0.15	0.31	483	−0.482	0.6
Fixed	Bedrock High Solute				0.35	0.11	484	3.32	0.001
Rand.	CT Valley – Intercept				−0.577				
Rand.	Berkshires – Intercept				0.577				

**Fig 3:**
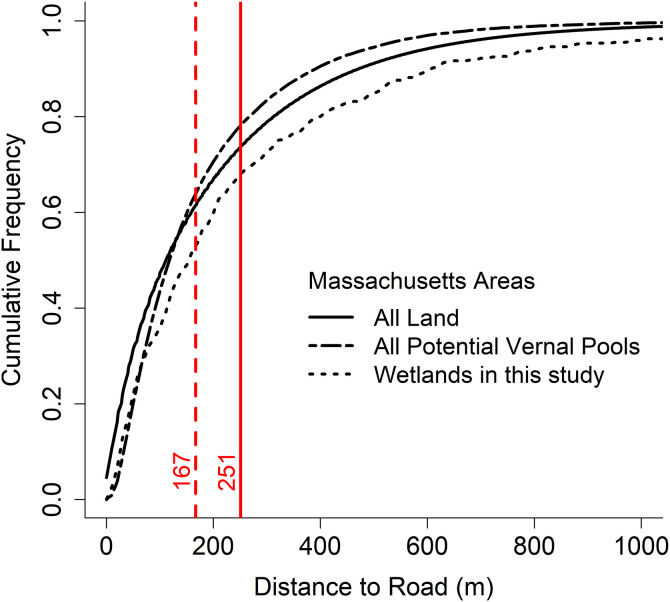
Cumulative distribution of distance to nearest road for all land in Massachusetts, all potential vernal pools (Burne 2001) in Massachusetts, and the wetlands sampled in our study. Vertical lines indicate the estimated end of the road salt zone, with standard error represented by vertical dotted line.

The mean conductivity for all wetlands beyond 251 m was 91 μS/cm (SD = 109 μS/cm), whereas conductivity was 168 μS/cm (SD = 180 μS/cm) between 167 m and 251 m, and 274 μS/cm (SD = 340 μS/cm) at wetlands within 167 m of roads. Between 150 m and 200 m, mean conductivity was 227 μS/cm (SD = 255 μS/cm). We also saw significant negative effects on water conductivity from high forest cover, high elevation, soil category 3 and low-solute category bedrock. When extrapolated across the entire state, our estimated potential salt effect zone from all roads would encompass 78% of potential vernal pools [34](Fig 3).

**Fig 4:**
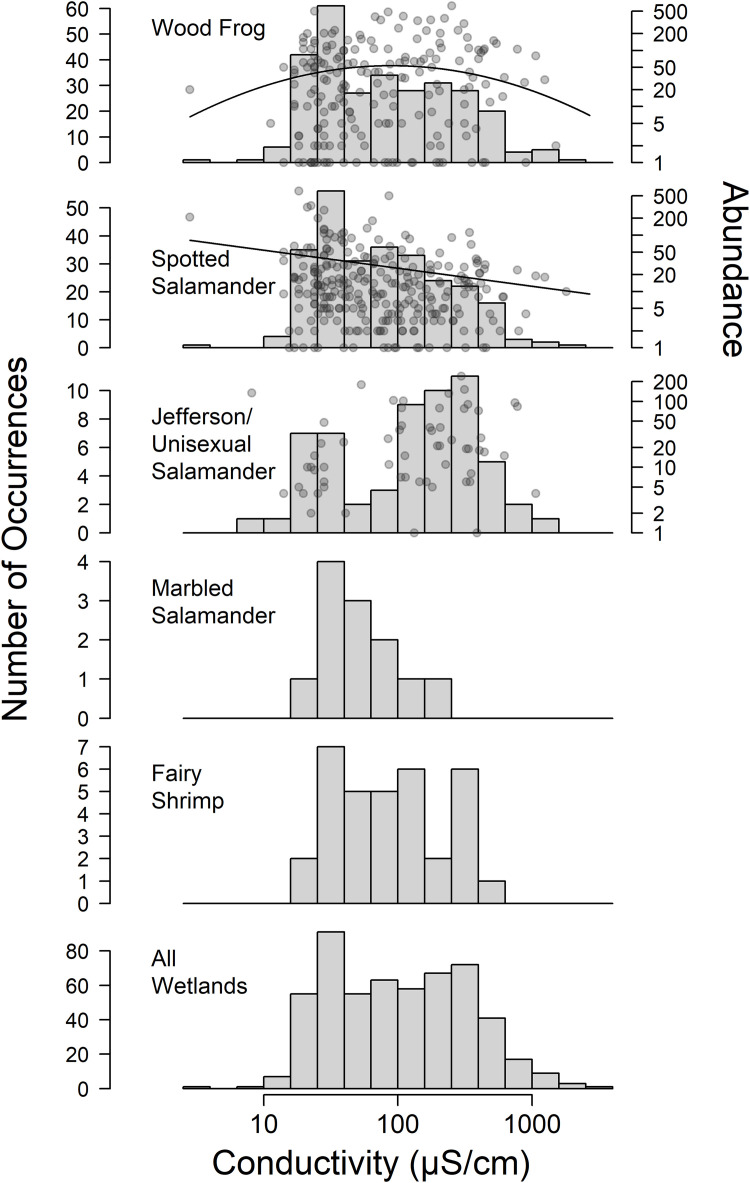
Distribution of conductivities (vertical bars) measured in wetlands where each of five vernal pool indicator taxa were observed during surveys of 541 wetlands across western Massachusetts, USA in 2008, 2009 and 2010. Points represent egg mass counts where available and the curves represent predicted egg mass abundance based on fitted N-mixture models.

We detected spotted salamanders at 264 ponds, wood frogs at 289 ponds, Jefferson salamanders at 59 ponds, marbled salamanders at 12 ponds and fairy shrimp at 34 ponds. The mean water conductivity was lower than the overall average of 200 μS/cm where we detected spotted salamanders (mean = 140 μS/cm, max = 1803 μS/cm), marbled salamanders (mean = 66 μS/cm, max = 190 μS/cm), wood frogs (mean = 160 μS/cm, max = 1802 μS/cm), and fairy shrimp (mean = 127 μS/cm, max = 406 μS/cm), and approximately the same for Jefferson salamanders (mean = 207 μS/cm, max = 1064 μS/cm; Fig 4). Where spotted salamanders were detected, we counted on average 29 egg masses (SD = 64) in wetlands of all conductivites and 17 egg masses (SD = 21) in wetlands with conductivity greater than 200 μS/cm. Where wood frogs were detected, we counted on average 45 egg masses (SD = 88) in wetlands of all conductivites and 50 egg masses (SD = 100) in wetlands with conductivity greater than 200 μS/cm. Where Jefferson salamanders were detected, we counted on average 39 egg masses (SD = 50) in wetlands of all conductivites and 52 egg masses (SD = 58) in wetlands with conductivity greater than 200 μS/cm.

In the occupancy model for spotted salamanders, including conductivity as a detection covariate did not substantially change model interpretation or other parameter estimates, this term was not statistically significant (p = 0.8), and resulted in a slightly higher AIC (ΔAIC = 1.9), so we selected this simpler model for analyses (Table 2). There was no evidence for lack of fit of spotted salamander occupancy in bootstrap simulations (p = 0.3). Conductivity had a statistically significant negative effect (p = 0.004) on occupancy in the final model.

**Table 2:** Parameter estimates from occupancy model for spotted salamander (*Ambystoma maculatum*) at 485 wetlands in western Massachusetts, USA sampled in spring of 2008, 2009, and 2010, with variables scaled prior to modeling using the original mean and standard deviations listed.

Parameter	Mean	SD	Units	Model	Estimate	SE	Z	*p*
Intercept				Occupancy	0.78	0.20	4.0	<< 0.001
log(Conductivity)	4.63	1.2	log(μS/cm)	Occupancy	−0.47	0.16	−2.9	0.004
log(Dist. from Road)	4.90	1.2	log(m)	Occupancy	0.15	0.17	0.86	0.4
Elevation	289	159	m	Occupancy	0.22	0.21	1.1	0.3
Perimeter	177	132	m	Occupancy	−0.16	0.14	−1.1	0.3
Canopy Cover	66	13	%	Occupancy	1.12	0.20	5.7	<< 0.001
Intercept				Detection	−14.8	3.3	−4.6	<< 0.001
Canopy Cover	66	13	%	Detection	−0.77	0.35	−2.2	0.03
Day of Year	112	13	Days	Detection	0.154	0.032	4.8	<< 0.001

In the wood frog occupancy model, although including conductivity as a detection covariate slightly reduced the model AIC (ΔAIC = −1.9) and this term was statistically significant (p = 0.002), the model intercept and associated uncertainty was extremely high (5.3, SE = 4.9) indicating model instability and an ecologically unrealistic fitted baseline occupancy of 99.5%, likely due to inability to partition the effects of conductivity between detection and occupancy. Removing conductivity from the detection model appeared to resolve this issue, resulting in a more reasonable fitted baseline occupancy of 82%. Although parameter values changed moderately between the two models, the ecological interpretations of their effects on occupancy did not and so we selected the more parsimonious, more stable, and more realistic model without conductivity as a detection covariate. The model did not show strong evidence of lack of fit (p = 0.3). Conductivity was inversely correlated with wood frog occupancy in the final model and this effect was statistically significant (p = 0.014; Table 3).

**Table 3:** Parameter estimates from occupancy model for wood frog (*Lithobates sylvaticus*) at 485 wetlands in western Massachusetts, USA sampled in spring of 2008, 2009, and 2010, with variables scaled prior to modeling using the original mean and standard deviations listed.

Parameter	Mean	SD	Units	Model	Estimate	SE	Z	*p*
Intercept				Occupancy	1.53	0.59	2.6	0.010
log(Conductivity)	4.63	1.2	log(μS/cm)	Occupancy	−0.84	0.34	−2.5	0.014
log(Dist. from Road)	4.90	1.2	log(m)	Occupancy	−0.14	0.28	−0.50	0.6
Elevation	289	159	m	Occupancy	0.72	0.43	1.7	0.09
Perimeter	177	132	m	Occupancy	−0.03	0.17	−0.17	0.19
Canopy Cover	66	15	%	Occupancy	0.33	0.25	1.3	0.9
Intercept				Detection	0.043	1.27	0.034	0.97
Canopy Cover	66	15	%	Detection	0.047	0.22	0.22	0.8
Day of Year	112	13	Days	Detection	0.008	0.012	0.64	0.5

For the N-mixture model of spotted salamander abundance, including a quadratic conductivity term increased AIC (ΔAIC = 2), whereas including conductivity as a detection covariate reduced AIC (ΔAIC = −1.8), so we selected the model with conductivity as a detection covariate but no quadratic conductivity term. This model showed signs of moderate overdispersion within an acceptable range for ecological data (ĉ = 1.8) [52], and showed no strong evidence for lack of fit (p = 0.24). After correcting for overdispersion, the model retained a significant negative effect of conductivity on abundance of spotted salamanders (p = 0.02), with predictions of spotted salamander abundance declining steadily with increasing conductivity (Table 4).

**Table 4:** Parameter estimates from N-mixture model for spotted salamander (*Ambystoma maculatum*) at 485 wetlands in western Massachusetts, USA sampled in spring of 2008, 2009, and 2010, with variables scaled prior to modeling using the original mean and standard deviations listed and with standard errors, z-values, and p-values adjusted for overdispersion.

Parameter	Mean	SD	Units	Model	Estimate	SE	Z	*P*
Intercept				Abundance	3.24	0.15	21	<< 0.001
log(Conductivity)	4.63	1.17	log(μS/cm)	Abundance	−0.38	0.17	−2.3	0.02
log(Dist. From Road)	4.9	1.23	log(m)	Abundance	0.13	0.14	0.94	0.4
Elevation	289	159	m	Abundance	0.02	0.15	0.15	0.9
Perimeter	177	132	m	Abundance	−0.21	0.16	−1.4	0.18
Canopy Cover	69	17.3	%	Abundance	0.10	0.18	0.55	0.6
Intercept				Detection	−3.35	0.98	−3.4	0.0006
log(Conductivity)	4.63	1.17	log(μS/cm)	Detection	−0.12	0.084	−1.4	0.17
Canopy Cover	69	17.3	%	Detection	0.85	0.11	7.6	<< 0.001
Day of Year	112	13.3	Days	Detection	0.0320	0.0064	5.0	<< 0.001

For the wood frog abundance model, including the quadratic conductivity term resulted in a lower AIC (ΔAIC = −1.3), as did inclusion of conductivity as a detection covariate (ΔAIC = −2.4), so we included both of these parameters. The model showed no strong evidence of lack of fit (p = 0.19) and had moderate but acceptable overdispersion (ĉ = 1.5), with both the linear conductivity term (p = 0.1) and the quadratic conductivity term (p = 0.1) approaching but not meeting statistical significance after correcting for overdispersion (Table 5). Based on parametric bootstrapping simulations of our model parameters, wood frogs were predicted to have a peak abundance at around 130 μS/cm but with large uncertainty in the location of the peak (SD = 280 μS/cm; Fig 4).

**Table 5:** Parameter estimates from N-mixture model for wood frog (*Lithobates sylvaticus*) at 485 wetlands in western Massachusetts, USA sampled in spring of 2008, 2009, and 2010, with variables scaled prior to modeling using the original mean and standard deviations listed and with standard errors, z-values, and p-values adjusted for overdispersion.

Parameter	Mean	SD	Units	Model	Estimate	SE	Z	*p*
Intercept				Abundance	3.74	0.18	21	<< 0.001
log(Conductivity)	4.63	1.17	log(μS/cm)	Abundance	1.85	1.18	1.6	0.12
log(Conductivity)^2^	22.8	11.2	log(μS/cm)^2^	Abundance	−1.97	1.19	−1.7	0.10
log(Dist. from Road)	4.9	1.23	log(m)	Abundance	0.01	0.20	0.068	0.9
Elevation	289	159	M	Abundance	0.40	0.21	1.9	0.06
Perimeter	177	132	M	Abundance	−0.15	0.23	−0.66	0.5
Canopy Cover	69	17.3	%	Abundance	−0.10	0.19	−0.53	0.6
Intercept				Detection	7.60	0.45	17	<< 0.001
log(Conductivity)	4.63	1.17	log(μS/cm)	Detection	−0.076	0.044	−1.7	0.09
Canopy Cover	69	17.3	%	Detection	0.254	0.027	9.5	<< 0.001
Day of Year	112	13.3	Days	Detection	−0.0662	0.0033	−20	<< 0.001

For wetlands with average values of all parameters except conductivity, the threefold higher conductivity observed in the estimated road effect zone translated to predicted 14% (SE = 5%) decline in occupancy of spotted salamanders, a predicted 15% (SE = 5%) decline in occupancy of wood frogs, 29% (SE = 11%) lower spotted salamander population sizes, and 19% (SE = 14%) lower wood frog population sizes.

## Discussion

Rather than remaining immediately adjacent to roadsides, our data suggest that deicing salt finds its way into isolated wetlands within a zone that extends at least 170 m—and likely as far as 250 m—into the surrounding landscape. This estimated potential road salt zone covers the majority of the lands and wetlands in our region (Fig 3) and is consistent with the findings of Karraker et al. [30], who found impacts of road salt in vernal pools up to 172 m from road edges in the Adirondack region of New York. This scale is also of the same magnitude as the annual migration distance for many vernal pool-dependent amphibians [53–55].

Here, we have modeled mean conductivity across hundreds of sites, whereas the chemistry of any particular wetland is controlled by myriad complex factors related to human decision making and environmental setting. For example, one wetland that occurred only 16 m from a road had a very low conductivity of 20 μS/cm; this road occurs in a state forest where the managers close that section of road in winter rather than applying deicing salt. Another road that occurs 89 m from a wetland that had a conductivity reading of only 3 μS/cm appears to be a dirt road on satellite imagery, and thus is unlikely to be treated with salt. The patterns observed at these two sites are consistent with our overall conclusion that elevated wetland conductivity is driven by road salt application. As an observational study, however, we cannot rule out the possibility that other hidden variables associated with roadways are driving the patterns in our data.

One potential confounding variable is land use history, the legacy of which can leave lasting signatures on an ecosystem [56]. Consider that 88% of our sites occurred on land classified as either forest (n = 295) or woody wetlands (n = 183) in 2008, but in 1830 during the peak of agriculture in Massachusetts, 71% of 344 sites with available data were mapped as either open lands (n = 236) or meadow (n = 8), including 61% of sites currently classified as forested [57]. Livestock manure is known to elevate salinity of local soil and groundwater [58,59], and high conductivities measured in forested wetlands today could conceivably be the result of manure left at sites 200 years ago. Open land in the 1830s is correlated with both distance from road and conductivity at our sites, and thus could suggest an alternative explanation of the correlation between distance from road and conductivity. However, when we examined a model of the sites with available 1830s data, distance from road remained an important predictor even with 1830s open space included as a covariate (S1 Appendix).

In the present day, although most of our sites are mapped as forested at the point locations, runoff from adjacent lands such as nearby farms, developments, or natural features may also contribute elevated salinity to our focal wetlands. For example, two wetlands in our study that lie 1060 m and 1260 m from the nearest road measured conductivities of 130 μS/cm and 113 μS/cm, respectively. Relative to other wetlands this far from roads, these readings appear high, however, both of these ponds are positioned immediately downslope from blocks of basalt bedrock which likely releases high levels of cations, elevating conductivity of those wetlands [60]. Similarly, two wetlands 760 m and 1060 m from the nearest road also have relatively high conductivities of 196 μS/cm and 330 μS/cm, respectively. Both of these wetlands appear to be hydrologically connected to much larger swamps that may transport ions from a variety of sources on the landscape of both non-human and human origin.

Without chemical analysis of the constituent ions causing elevated conductivity in our wetlands, our study in isolation cannot definitively trace the origins back to road salt—even if the ions originated from roads, it is conceivable that other chemicals associated with roadways, such as roadside fertilizers, metals released from vehicles, leaching from road construction materials, or other pollutants could be the source of elevated conductivities. However, viewing this study in context, the most parsimonious explanation for elevated conductivity near roadways is the influence of deicing salts. The Massachusetts Department of Transportation (MassDOT) applies roughly 500,000 tons of deicing salts to roadways annually primarily as forms of sodium chloride and, beginning mostly after 2011, magnesium chloride, and this figure does not include the other 80% of roads not managed by MassDOT [61]. Chloride ions dissolved from road salt have been linked to higher conductivity in freshwater lakes and rivers in many settings, with winter pulses in chloride concentrations coinciding with the seasonal application of road salt [62–65]. We interpret our data as an extension of this well-documented pattern to isolated vernal pools where there have been fewer studies on anthropogenic salinization, but where Karraker [30], in a similar landscape study near our region linked conductivity in vernal pools to sodium and chloride concentrations attributed to road salt.

Road salt moves through ecosystems via both surface and groundwater flow [66]. Point locations used for wetlands in this study are based on where along the perimeter we took conductivity readings, whereas parts of the larger wetlands in our study may have approached much closer to roadways, facilitating spread of salt through mixing of water within the body itself. This may explain why the smallest wetlands in our study only appeared to be affected by salt within a zone closer to roadways. These smaller wetlands may also have smaller watersheds intersecting fewer roads and fewer connections to flowing surface and ground water that may transport salt to the wetlands.

In our data, mean elevated conductivity near roads predicted lower detection rates for spotted salamanders, even after accounting for direct effects of proximity to roads and other environmental factors. Similarly, we found that high conductivities reduced egg mass counts, which is consistent with other studies showing that elevated salinity in roadside wetlands can reduce embryonic and larval survival [22,30,67], potentially causing these wetlands to function as sink populations. Although there is large uncertainty in our parameter estimates for wood frog abundance, a unimodal relationship between conductivity and wood frog abundance is consistent with prior studies; on the low end of water conductivity, wood frog tadpole abundance is positively correlated with increasing dissolved solutes such as nitrogen and phosphorous which drive productivity and thus boost food availability, whereas at high concentrations, dissolved solutes—particularly chlorine and sodium which are the primary constituents of road salt—become toxic [68,69]. Other studies have shown evidence that local populations can partially adapt to better tolerate elevated salinity, potentially offsetting some of the population impacts [70–72]. However, road salt has been shown to have a large variety of cascading ecosystem impacts that are cause for much concern [73–75].

The correlations between conductivity and amphibian populations should be interpreted cautiously. In particular, the positive association between conductivity and Jefferson salamander raw egg mass counts was based on too few points for full statistical analysis and should not be taken as indicating tolerance or preference. Similarly, the results for wood frog models showed mixed results, with an initially unstable occupancy model when conductivity was included as a detection covariate and lack of statistical significance in the abundance model with high uncertainty in parameter estimates and predictions. We also cannot rule out the possibility that the observed amphibian patterns and conductivity are both responding to hydroperiod or other unmeasured hydrologic variables [76], rather than salinity controlling amphibian communities directly. Due to canopy cover obscuring aerial views of northeastern vernal pools, currently the only way to assess hydroperiod is via ground-based measurements which are not practical at the scale of this study. However, emerging GIS technology [77] and approaches [78] may provide readily available hydroperiod data in the coming years and we recommend revisiting studies such as ours as these data become available.

Our measures of population size should be considered coarse indices to abundance; they were based on visual surveys during daytime perimeter walks that may have missed many egg masses farther from the edges. Karraker et al. [30] constructed demographic models to examine how conductivity affects population sizes, finding that at 500 μS/cm, depending on the degree of larval density dependence, spotted salamander populations may be reduced by anywhere from 40% to over 70% and wood frog populations may be reduced by anywhere from 15% to over 70%. In our study, 14% of wetlands between 50 m and 167 m from roads had conductivities greater than 500 μS/cm, and we expect these numbers to grow in the future with the anticipated continued salinization of freshwater systems [63]. While not all populations within the salt effect zone will experience elevated conductivities or resultant population declines, management of a sensitive wetland population should not be assumed to be safe from the impacts of road salt, even if the wetland occurs 100 m or more from the nearest road.

Application of road salt is ongoing. The decisions concerning when, where, and how much salt to apply to roads each winter are made every winter by various jurisdiction-dependent roadway management authorities. Anecdotally, we have observed that differences in the decision-making process can result in dramatic differences in the intensity of salt application on a single rural road when crossing town lines in our region. According to Massachusetts GIS data, 64% of the nearest roads to our wetlands are owned by local towns or municipalities, 9% are owned by the state Department of Transportation, 1% are owned by state conservation or recreation agencies and 26% are “unaccepted” roads that are likely privately managed. Thus, road salt application strategies are largely in the hands of local decision-makers, who may also be the ones best positioned to balance local conservation and road safety concerns. Although we have estimated the size of the mean potential salt effect zone across our entire region, this is arrived at by averaging across substantial variation. Each wetland has its own idiosyncratic relationship to the surrounding environment and thus conservation decisions for any particular site should incorporate the specific local context. More nuanced management guidance requires further work on how the flow of road salt through the environment is controlled.

While northeastern U.S. forests have recovered over the past half century, environmental pollution from road salt application has worsened [79]. With the salinity of freshwater bodies increasing [65], as salts continue to accumulate in the future, we can expect increasing impacts to wetland communities. Rapid salt accumulation may be particularly likely in isolated wetlands such as vernal pools. Vernal pools are typically not connected to flowing surface waters and are often perched above ground water [31]. Evapotranspiration tends to be an important component of water loss from vernal pools, and thus salt may accumulate more rapidly in vernal pools than in lakes or streams with permanent outputs of flowing water.

Given the prospects of continued salt accumulation wherever deicing chemicals are applied, it is essential that we consider ways to balance public safety needs with the long-term environmental costs of road maintenance. Possible strategies include reducing the amount of salt applied, relying on better plows in lieu of salt, utilizing alternative deicing chemicals, and minimizing runoff through structural features at road margins [13,14,80–83].

As we consider the ongoing application of salt on our roadways, it would be informative to layer social and political variables, such as town-by-town differences in road salt application, on top of our data to understand opportunities for changing salt application. Now that 15 years have elapsed since we first collected our data, it would be further informative to revisit the same ponds to see if salinity has continued to increase within the salt-effect zone relative to those beyond the zone.

## Supporting information

S1 Appendix Underlying data, R code, and all outputs of models associated with this manuscript. (ZIP)
