# Supplementary figures and images for: The reach of road salt into vernal pools and the response of amphibians
Source: PLoS One. 2025 Oct 23;20(10):e0329680. doi: 10.1371/journal.pone.0329680 (PMC12548906; doi:10.1371/journal.pone.0329680)

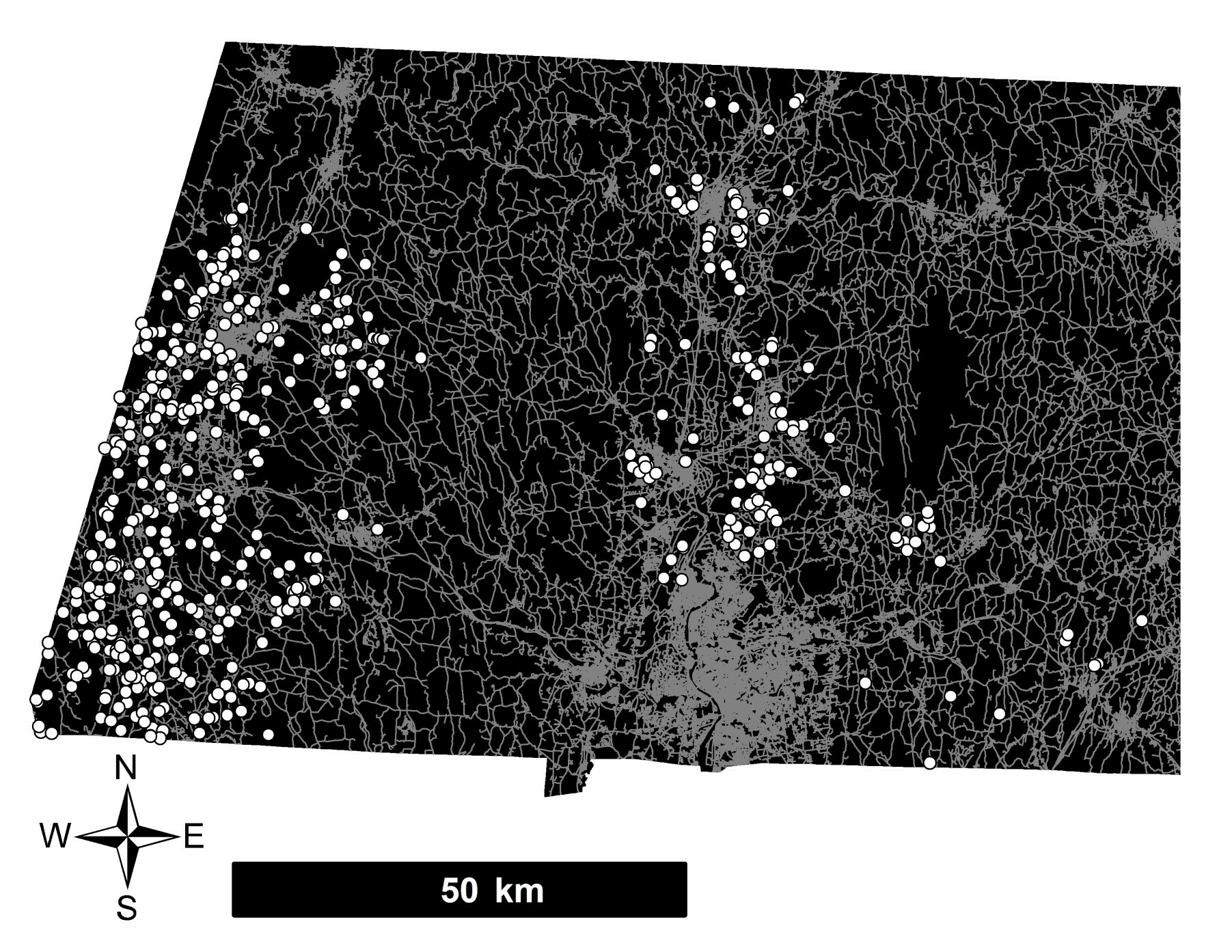

Supplement: S1 Appendix — (ZIP) [file pone.0329680.s001.zip › Fig1.tif]

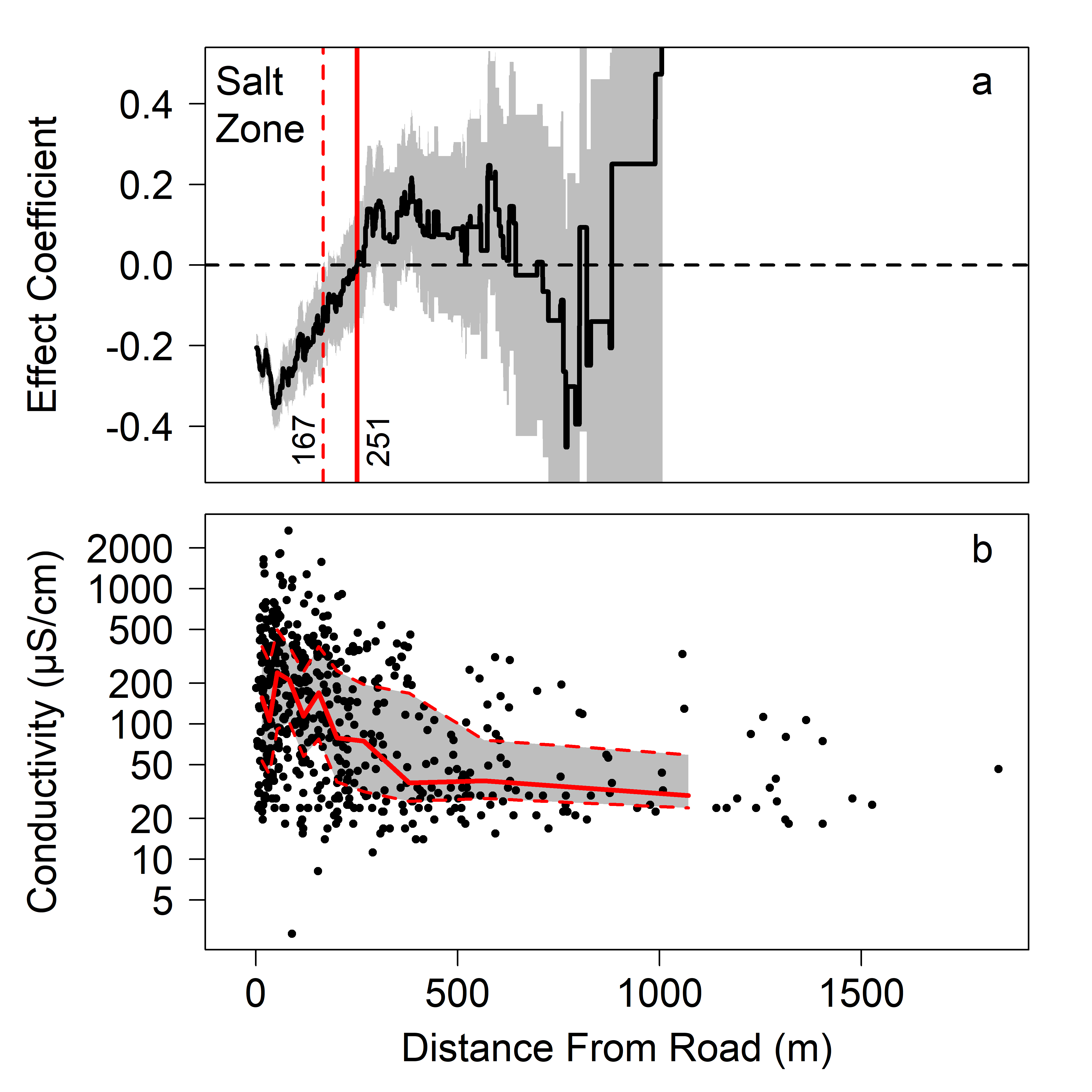

Supplement: S1 Appendix — (ZIP) [file pone.0329680.s001.zip › Fig2.tif]

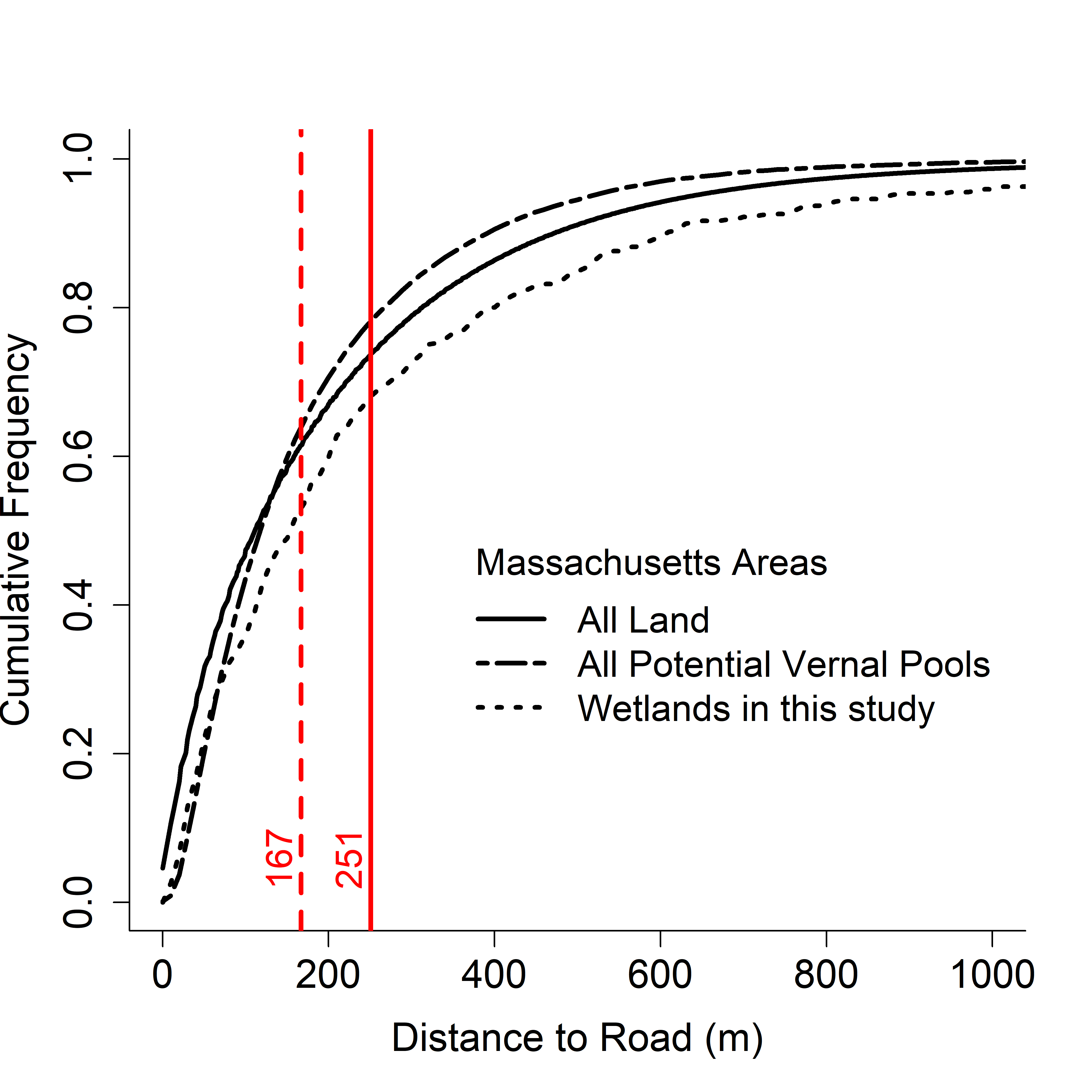

Supplement: S1 Appendix — (ZIP) [file pone.0329680.s001.zip › Fig3.tif]

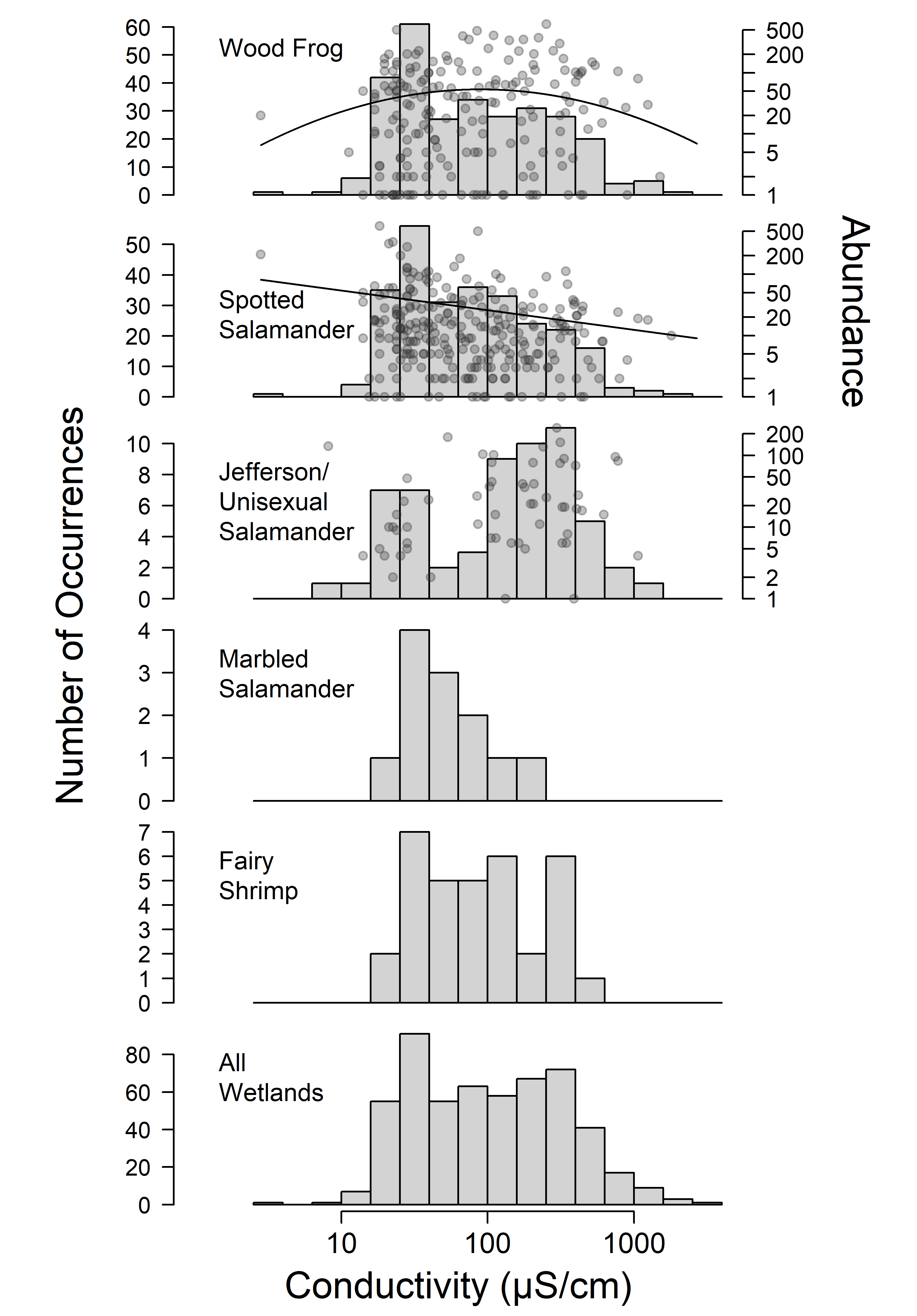

Supplement: S1 Appendix — (ZIP) [file pone.0329680.s001.zip › Fig4.tif]

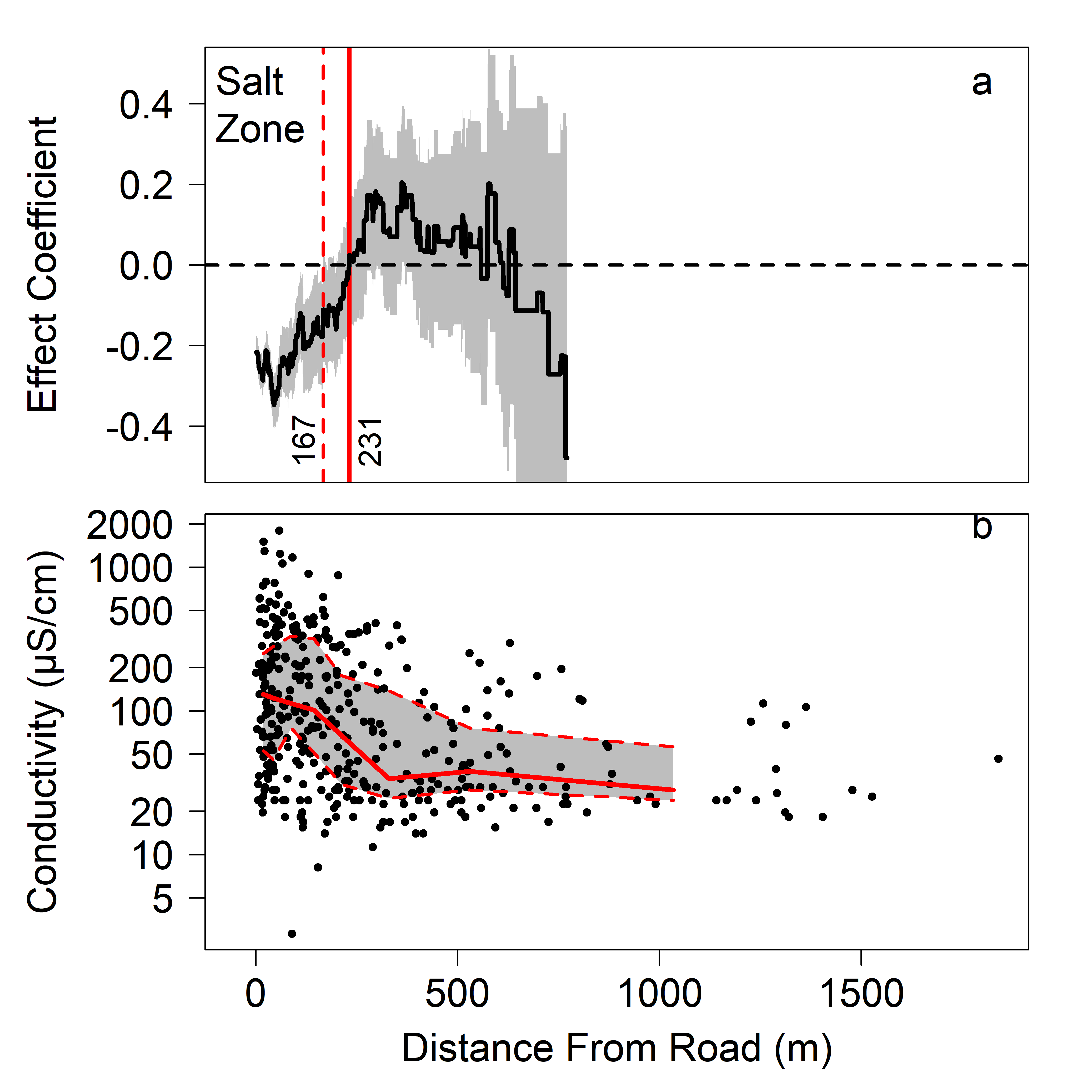

Supplement: S1 Appendix — (ZIP) [file pone.0329680.s001.zip › only_VPindicators_pond_salt_zone.tif]
