# Supplementary material for: The reach of road salt into vernal pools and the response of amphibians
Source: PLoS One. 2025 Oct 23;20(10):e0329680. doi: 10.1371/journal.pone.0329680 (PMC12548906; doi:10.1371/journal.pone.0329680)
Supplement: Attachment — Submitted filename: line-by-line_revision1_V1.pdf [file pone.0329680.s003.pdf]

#	Comment	Response
ed-1	Please ensure that your manuscript meets PLOS ONE's style requirements, including those for file naming.	We have checked to the best of our ability, please let us know if something is wrong.
ed-2	In your Methods section, please provide additional information regarding the permits you obtained for the work. Please ensure you have included the full name of the authority that approved the field site access and, if no permits were required, a brief statement explaining why.	Added sentence to Field Data Collection section of Methods: "***No permits were necessary for this work, as it did not require handling organisms, we obeyed all posted signs, and Massachusetts law generally allows public access to private or public land that is not legally posted (Massachusetts G.L. c. 266 § 120: Trespass).***"
ed-3	Thank you for stating the following financial disclosure: [The Natural Heritage & Endangered Species Program of the Massachusetts Division of Fisheries and Wildlife; the National Science Foundation Graduate Research Fellowship Program; The Amherst College Lloyd I Rosenblum Memorial Fellowship Program; McIntire-Stennis funding, project award no. ME0-42312, from the U.S. Department of Agriculture's National Institute of Food and Agriculture.]. Please state what role the funders took in the study. If the funders had no role, please state: "The funders had no role in study design, data collection and analysis, decision to publish, or preparation of the manuscript." If this statement is not correct you must amend it as needed. Please include this amended Role of Funder statement in your cover letter; we will change the online submission form on your behalf.	Added sentence as suggested to Acknowledgments.

#	Comment	Response
ed-4	When completing the data availability statement of the submission form, you indicated that you will make your data available on acceptance. We strongly recommend all authors decide on a data sharing plan before acceptance, as the process can be lengthy and hold up publication timelines. Please note that, though access restrictions are acceptable now, your entire data will need to be made freely accessible if your manuscript is accepted for publication. This policy applies to all data except where public deposition would breach compliance with the protocol approved by your research ethics board. If you are unable to adhere to our open data policy, please kindly revise your statement to explain your reasoning and we will seek the editor's input on an exemption. Please be assured that, once you have provided your new statement, the assessment of your exemption will not hold up the peer review process.	Our commitment to data publishing remains unchanged, as written in our MS, "*OPEN RESEARCH STATEMENT: R scripts and underlying data for reproducing these analyses will be archived on Figshare, with the exception of the data for sensitive species protected under the Massachusetts Endangered Species Act. For data associated with those species, contact the Natural Heritage and Endangered Species Program of the Massachusetts Division of Fisheries and Wildlife.* " Note that we have already uploaded data onto Figshare for the purposes of review. If this is insufficient, please explain what is missing.
ed-5	Please include a caption for figure 4.	All figures now should have captions.
R1-1	•First of all, I suggest change title – there are two main topics in manuscript – salinization of wetland at difference length from road and its impact on amphibian population – title should include both topics. Off course you mention also about shrimps, if title would not be long you can add it too.	Changed title to: "*The reach of road salt into vernal pools and the response of amphibians* "
R1-2	Secondly, you results presented half to half information about salinity vs road distant, and salinity vs amphibians – but you only present on figures first one. You would be very valuable to present some more figures/tables with salinity vs amphibians analyses, including some statistical. You make huge work to investigate such a number of wetlands – it should be a lot data base to make some statistic analyses.	Thank you for this suggestion, we have now added a Figure 4, which is a set of histograms, scatterplots, and fitted model predictions showing the distribution of conductivities across the different wetlands where focal taxa were detected. We have also added several new sentences to the fourth paragraph of the Results providing summary statistics on the relationship between species and conductivity. Finally, we have provided four supplemental tables in "S1 Tables" that give the parameterizations of the Occupancy and N-Mixture model fits for amphibian occurrence and abundance.

#	Comment	Response
R1-3	You collected data almost 15 years ago. It no bad, because they are still give new knowledge, but you discussion part (or rather the entire manuscript) compare you research with newer publications (only 7 from 45 are published after 2015). There are many new publication and some older publication, about relation of salinization and amphibians, like for example: https://doi.org/10.1890/ES14-00534.1; https://doi.org/10.1007/s00442-022-05243-3; https://doi.org/10.1002/ece3.11069; https://doi.org/10.1643/h2020070; https://doi.org/10.1016/j.envpol.2016.11.060; https://doi.org/10.1080/02705060.2009.9664301	Thanks for these suggestions, these are great studies. We have added all the suggested references (we had already cited Jones et al 2017 in first submission, but we added another sentence about this work in the revision)
R1-4	In Material and Methods you give information about number of days with max temperature below 0 °C, but road salt is also use when max is above 0 °C, and for night temperature falling below this value. Please add this information, which is very important in relation to road salt application	There seems to be some confusion caused by our initial sentence, so we have decided to just delete the portion of the sentence in question, as it was an imperfect and possibly misleading metric.
R1-5	There is no information about wetland base characteristic – area – you are using GIS, so it should not be a problem to add this.	We do already include perimeter in the first submission -- see methods, results, and discussion -- measured in the field to get at wetland size in our analyses. At the reviewer's suggestion, we have also included a measure of surface area by combining perimeter with shape complexity measured in the field. The reviewer's suggestion of using GIS, while a good idea, is not possible. Most of these are vernal pools which are too small and ephemeral to be mapped on GIS layers, so we cannot extract wetland area except for during our field visits. We do have a pending NASA grant using newer satellites to derive area, so maybe in five years this would be possible. Note, that in responding to this, we found a handful of missing perimeter values entered as "0" instead of NA, and thus re-ran all models that previously included this parameter - nothing of substance changed, but you will notice minor shifts in the last digit on some values reported in the Results due to this.

#	Comment	Response
R1-6	Wetland area is important in relation with TDS measurement – if wetland are small, you can measure in one place, but what if they are large? It important, because as you wrote about different hydrological connection of wetlands. If all were similar you can measure for example at inflow part. But for now this information is missed in manuscript.	Yes, we agree that there is an issue relating wetland size to where we ended up measuring conductivity; we wrote about this in the second paragraph of the Discussion in the original submission. At present time, we do not have any way to quantify hydrological connections for these wetlands. These wetlands are largely vernal pools, which are nominaly not connected to any other surface waters, and typically quite small, with little hydrologic connection to other waters, see Brooks 2005 about the complexity in modeling vernal pool hydrology. We would be happy to elaborate on this point if there is anything more the reviewer would like us to discuss.
R1-7	Related to above, similar question about amphibian and shrimp investigation – on which part of wetland you analyse them	Please see the methods, middle of first paragraph of Field Collection, "*We walked the entire perimeter of each wetland inspecting for signs of wood frogs, spotted salamanders, Jefferson salamanders, marbled salamanders, and fairy shrimp.* " Because we walked the entire pond perimiter searching for species, there is not as much potential for bias as in the conductivity measures. It is true that one could imagine that in larger wetlands it would be easier to miss egg masses in the centers, however we did not see a correlation between pond size and detection error.
R1-8	Finally, you collect a lot of data, but you don't meet Plos One standards - "The PLOS Data policy requires authors to make all data underlying the findings described in their manuscript fully available without restriction, with rare exception (please refer to the Data Availability Statement in the manuscript PDF file). The data should be provided as part of the manuscript or its supporting information, or deposited to a public repository. For example, in addition to summary statistics, the data points behind means, medians and variance measures should be available. If there are restrictions on publicly sharing data—e.g. participant privacy or use of data from a third party—those must be specified."	We completely agree with the open data standards -- that is why we uploaded our underlying data (including wetland survey results and geospatial layers) and R scripts that are fully executable with the provided data to FigShare with our first submission, linked in our coverletter, as per PLOS policy (and plan to permanently archive all data on Dryad). It appears the reviewer must have missed this link somehow, and I hope they are able to find it with the resubmission. The one caveat is that we did not provide the location of rare species protected under state code which we were contractually obliged to keep from public view, however, these data are available upon request from the state agency that maintains rare species records. If there is some other data the reviewer has in mind, please let us know, happy to provide.

#	Comment	Response
R2-1	It is well-recognized that detection of amphibian use of wetlands is subject to false negatives (lack of detection when the wetland is actually used). The authors seem to collect data that might let them estimate detection probabilities as 51 wetlands were visited two years in a row. It is unclear why the authors made no attempt to include detection errors in their modeling efforts. Concerns around detection error are particularly concerning for explosive breeders such as wood frogs. On a related note, the authors should also consider date of visit in their analyses for both conductivity and amphibian occurrence as detection probabilities and road salt effects are expected to diminish later in spring and	Given that the reviewer was not satisfied with our handling of detection error outside of the formal model, we now explicitly incorportate detection error within a formal occupancy framework as suggested, for both the occupancy and abundance (N-Mixture) components of the manuscript. At the reviewer's suggestion, we also include day of year in the amphibian models, but it made for worse AIC in the model of conductivity (see new sentence in Methods:Road Effect Zone paragraph on this)
R2-2	Wetland hydroperiod has been well established as a primary controller of community structure in vernal pool and other depression wetland systems. It seems to draw any conclusion concerning the effects of road salts on amphibian use of wetland, the authors would need to include some measures of hydroperiod as a covariate in any of their analyses.	Yes, hydroperiod is important. But, beyond the fact that these were all identified as seasonal vernal pools in the 1990s, we do not have hydroperiod data -- this is notoriously difficult to predict without in-situ measurments, which are costly for large scale studies such as ours with hundreds of wetlands. It is true that newer satellite data can attempt to get at this, but this is an emerging technology beyond our scope here (we do have a pending NASA grant to do this using the soon-to-launch NISAR satellite, perhaps in 5 years this will be possible). We still feel these data are worth presenting and have added a new paragraph in the discussion acknowledging the reviewer's important caveat: "***We also cannot rule out the possibility that the observed amphibian response and conductivity are both responding to hydroperiod or other unmeasured hydrologic variables (e.g. Waterkeyn et al. 2008), rather than salinity controlling amphibian communities directly. Due to canopy cover obscuring aerial views of northeastern vernal pools, currently the only way to assess hydroperiod is via ground-based measurements which are not practical at the scale of this study. However, emerging GIS technology (e.g. Kellogg et al. 2020) and approaches (e.g. Bourgeau et al. 2016) may provide readily available hydroperiod data in the coming years and we recommend revisiting studies such as ours as these data become available.*** "

#	Comment	Response
R2-3	Lines 106-107: I am not familiar with this approach. The authors convert TDS to conductivity by simply dividing by 0.71. There is no information presented on this relationship or its relationship to ions associated with road salts.	Thank you for this question, we have added a citation into the Methods section to a review of the relationship between "TDS" and conductivity (Rusydi 2017) to the sentence in question. Field sensors such as the one we used cannot directly measure "TDS;" instead they have electrodes in the water and measure the current passing between the electrodes, i.e. conductivity. Thus, even though the sensor reports "total dissolved solutes," that is not really what it measured. TDS is extremely difficult to quantify in the field, so it has long been standard practice to assume a linear relationship between TDS and conductivity, however the relationship is not actually so simple . Therefore, we reported what the instrument actually measured -- conductivity -- not the artificial estimate it made of TDS. This is standard practice in our field (e.g. see Karraker 2008)
R2-4	With no information on the relationship between Real Salt associated ions and TDS it is impossible to know that TDS is measuring road salt impacts.	We believe the reviewer may be referring to other pollutants than deicing salts that could be causing the elevated conductivities we observed near roads. Assuming so, we have added a sentence and modified another sentence with citation in the Discussion. However, please let us know if we have misinterpreted this comment. These sentences now read: "***It is also possible that other chemicals associated with roadways, such as fertilizers, metals released from vehicles, leaching from road construction materials, or other pollutants could be the source of elevated conductivities. However, the conclusion that road deicing salt itself is primarily responsible for the elevated conductance readings is consistent with our understanding of the system, consistent with previous literature, and is the most parsimonious explanation available (Kaushal et al. 2005).*** "
R2-5	It is not clear why the authors made no attempt to include detection probabilities in their analyses of amphibian occurrence. The amphibians that were included in the study are seasonal and explosive breeders, making false negatives a real possibility. The authors did visit 51 wetlands twice, so they have the data to estimate detection probabilities and correct estimates of occurrence accordingly. However, they assume these data are 100% accurate and analyze transition probabilities. This assumption makes it hard to draw any conclusions concerning impacts distances.	Please see comment R2-1, we now take an occupancy approach as suggested.

#	Comment	Response
R2-6	On another but related note, the authors should use day of year in their analyses of both conductivity and amphibian occurrence as they are both expected to decline later in spring and summer.	For conductivity, we took the reviewer's suggestion and examined models including day of year, however, this was not correlated with conductivity and the AIC for those models was worse than the selected model -- see new sentences in revised MS: "***We also examined models post hoc containing additional covariates of wetland perimeter, wetland area, and day of year, but the model we chose a priori had the lowest AICc out of all possible parameter combinations examined using the dredge() function in the MuMIn package of R (Bartoń 2024)."***
R2-7	Lines 133-134: The authors note they transformed variables to assure normality. They do not appear to have checked for normality of conductivity in the regression models.	Please clarify -- yes, we did check for normality, that is why we log-transformed conductivity and distance from road, as stated in lines 133-134 of the original MS as the reviewer notes: "**To obtain normality, we log-transformed water conductivity and the distance to nearest road prior to regression analyses.**" Log-transforming led to much more normal distributions. It feels like there is a typo in the reviewer comment or some other information we are missing to fully understand the issue.
R2-8	Moreover, independent variables such as distance from roads are transformed to achieve linearity. Therefore, most important here is the effects of this transformation on their estimate of distance effects. How would their results change if they did not log-transform distance from roads?	Raw distance from road is a highly skewed variable and log-transforming does achieve normality, and, as noted by the reviewer, moves us closer to a linear relationship between response and predictors since we expect salt concentrations to decay exponentially with distance from road. This log transformation of distance from road reduces AIC by -11.9 in the full model, so appears to be the correct choice. At the reviewer's suggestion, we re-ran the analysis with distance from road not log-transformed to see if it would make a difference. It did not change where the mean estimate crossed the zero line in our model (251 meters), but it did reduce where the standard error crossed the line (153 m), thus slightly increasing the uncertainty of the road-effect zone. Note, that the reason this has minimal effect on our results is because our estimate of the zone distance is fundamentally non-parametric (iteratively adjusting the threshold distance and re-running the underlying parametric model). We added this new sentence to the Results: "***The choice to log-transform the conductivity response did not change the mean estimate of the salt effect zone, but when conductivity was not log-transformed, the uncertainty was higher as a result of poorer-fitting models.*** "

#	Comment	Response
R2-9	There is confusion on model description throughout the manuscript. On lines 138-140 and again on lines 149-151 the authors descript a series of independent variables, including distance from roads, as random effects and region as a fixed effect. This should be the other way around with region as the random effect and the other variables as fixed (because we expect for example that TDS will decline with distance from roads).	Good catch, we fixed this typo
R2-10	Moreover, in table 1 the fixed and random effects appear to be describe correctly, but the legend indicates a logistic regression when this is clear results from a linear	Typo fixed.
R2-11	Lines 158-159: egg mass counts would be more appropriately analyzed with a poisson error term.	This is correct, we would need to specify a poisson error term in the linear regression approach, however, we are now using an N-Mixture model which already assumes a poisson distribution
R2-12	Lines 184-185: This seems to be too simple an approach. I assume this is simply a spatial analysis conducted by buffering roads. There are wetlands well inside of the estimated effect zone that have the lowest conductivities while there are wetlands well outside of the effect zone that have conductivities above 200 microS/cm. What is really interesting here are these outliers. What cause them-could it be road type or traffic volume? We know that road salt effects decline with distance from roads. The next step should be figuring out what causes variation in effect distances and how they relate to things we can manage. I think this paper could be much more interests if information on road characteristics were included in some way.	Yes, this would be a great study! There are lots of directions to go with this analysis. But that is a different paper the reviewer is proposing. And, in fact, once our paper is published other researchers can use our data to perform just those analyses! Our goal here is to provide a simple first-pass look for decision-makers to understand the impacts of road salt application and base starting guidelines for policy. In this context, we are considering the "average" here, which of course means some ponds will be above and some below. We did add to the last paragraph of the MS further suggestions for a follow-up-study along the lines the reviewer suggested by looking at town-by-town differences in road salt application.
