# Supplementary material for: The reach of road salt into vernal pools and the response of amphibians
Source: PLoS One. 2025 Oct 23;20(10):e0329680. doi: 10.1371/journal.pone.0329680 (PMC12548906; doi:10.1371/journal.pone.0329680)
Supplement: Attachment — Submitted filename: vpSalt_SecondReviewerComments_v5.pdf [file pone.0329680.s004.pdf]

| # | Comment | Response |
|---|---|---|
| ed-1 | The abstract summarizes the main findings clearly and concisely. However, the abstract should clarify that the amphibian models focused only on species with sufficient data (e.g., spotted salamanders and wood frogs), and that occupancy modeling was not extended to all taxa mentioned. | The abstract currently says "Compared to average wetlands, the mean conductivity was lower in wetlands with marbled salamanders (Ambystoma opacum) and fairy shrimp (Eubranchipus sp.) and approximately the same for Jefferson salamanders (Ambystoma jeffersonianum and associated unisexual Ambystoma)***, but data for these species were insufficient for formal occupancy modeling.*** " Please let us know if more is needed here, thanks! |
| ed-2 | Potentially controversial claim – salt as sole conductivity driver. The manuscript implies that elevated conductivity is predominantly due to road salt. While plausible, this assumption could be overstated. The authors acknowledge other potential sources (fertilizers, metals, etc.) in the discussion but minimize them with a reference to parsimony and prior literature. Yet, no ion-specific measurements (e.g., chloride, sodium) are presented. Including some ion analysis, or at least referencing more detailed studies linking specific ion concentrations to road salt, would strengthen this assertion. | Added more citations into this paragraph, which has been filled out with many more sentences:"***Without chemical analysis of the constituent ions causing elevated conductivity in our wetlands, our study in isolation cannot definitively trace the origins back to road salt – even if the ions originated from roads, it is conceivable that other chemicals associated with roadways, such as roadside fertilizers, metals released from vehicles, leaching from road construction materials, or other pollutants could be the source of elevated conductivities. However, viewing this study in context, the most parsimonious explanation for elevated conductivity near roadways is the influence of deicing salts. Massachusetts applies roughly 500,000 tons of deicing salts to roadways annually primarily as forms of sodium chloride and, beginning mostly after 2011, magnesium chloride [61]. Chloride ions dissolved from road salt have been linked to higher conductivity in freshwater lakes and rivers in many settings, with winter pulses in chloride concentrations coinciding with the seasonal application of road salt [62–65]. We interpret our data as an extension of this well-documented pattern to isolated vernal pools where there have been fewer studies on anthropogenic salinization, but where Karraker [30], in a similar landscape study near our region linked conductivity in vernal pools to sodium and chloride concentrations attributed to road salt.*** " |
| ed-3 | Justify the 167–251 m threshold as ecologically meaningful or offer a sensitivity analysis using alternative definitions (e.g., specific conductivity thresholds, modeled change points, or species response breakpoints). | We have added a sentence in the Discussion:"***This scale is also of the same magnitude as the annual migration distance for many vernal pool-dependent amphibians[53–55].*** " Also, see the sentences later in the discussion: "***Karraker et al. [30] constructed demographic models to examine how conductivity affects population sizes, finding that at 500 µS/cm, depending on the degree of larval density dependence, spotted salamander populations may be reduced by anywhere from 40% to over 70% and wood frog populations may be reduced by anywhere from 15% to over 70%. In our study, 14% of wetlands between 50 m and 167 m from roads had conductivities greater than 500 µS/cm.*** " Taken together, these show that the spatial scale of the salt zone and the effect size of increased conductivity that we found in our study are both ecologically meaningful. If more is needed here, please clarify what other information we may provide. |

| # | Comment | Response |
|---|---|---|
| ed-4 | Consider including NLCD land cover type as a categorical predictor or discussing in greater detail how omitted variables might bias estimates. | Based on this suggestion, we examined NLCD land cover type as a predictor but it did not add much inference, see new addition to Methods: "***and the National Land Cover Database 2008 land cover type occurring at the wetland coordinates.*** " Perhaps the lack of importance is because the vast majority of the sites occurred in areas currently forested. So, we have now also included a section in the discussion that discusses historic land cover from the 1830s, which was only available for a subset of the data: "***Consider that 88% of our sites occurred on land classified as either forest (n = 295) or woody wetlands (n = 183) in 2008, but in 1830 during the peak of agriculture in Massachusetts, 71% of 344 sites with available data were mapped as either open lands (n=236) or meadow (n = 8), including 61% of sites currently classified as forested [57]. Livestock manure is known to elevate salinity of local soil and groundwater [58,59], and high conductivities measured in forested wetlands today could conceivably be the result of manure left at sites 200 years ago. Open land in the 1830s is correlated with both distance from road and conductivity at our sites, and thus could suggest an alternative explanation of the correlation between distance from road and conductivity. However, when we examined a model of the sites with available 1830s data, distance from road remained an important predictor even with 1830s open space included as a covariate.*** " |

| # | Comment | Response |
|---|---|---|
| ed-5 | The wood frog occupancy model did not find a significant effect of conductivity, yet strong inferences are made in the discussion based on abundance models. These interpretations could mislead readers into assuming stronger evidence of effect than warranted. Tone down conclusions related to wood frog occupancy and clarify that the effect was detected only in abundance and with uncertainty in peak location (68 µS/cm). | Good point. Note, that after examining models more closely, we found that the wood frog occupancy model was mis-specified causing instability and is now performing better (see new sentences in Results for details), but after adjusting for overdispersion (in response to comment R2-4), the abundance model terms are not considered significant. The bottom line, and the good point made here about emphasizing uncertainty and caution remain the same, and we have addressed this in two ways:<br><br>1. We have changed the wording around these results to add more nuance and caution. In the abstract: "...although the wood frog abundance model did not meet the threshold for statistical significance" and in the Discussion 7th paragraph here: "***Although there is large uncertainty in our parameter estimates for wood frog abundance, a unimodal relationship between...*** " and Discussion 8th paragraph here: "***The correlations between conductivity and amphibian populations should be interpreted cautiously... Similarly, the results for wood frog models showed mixed results, with an initially unstable occupancy model when conductivity was included as a detection covariate and lack of statistical significance in the abundance model with high uncertainty in parameter estimates and predictions."***<br><br>2. We have re-estimated the woodfrog predicted peak and included uncertainy through parametric bootstrapping, and emphasized the uncertainty in this peak in the new version: "***Based on parametric bootstrapping simulations of our model parameters, wood frogs were predicted to have a peak abundance at around 130 µS/cm but with large uncertainty in the location of the peak (SD = 280 µS/cm; Fig 4).*** " |

| # | Comment | Response |
| --- | --- | --- |
| ed-6 | Some additional key recent works could enhance the discussion, especially regarding ion-specific toxicity and landscape modeling of salt impacts.<br><br>References to be reflected:<br><br>Corsi, et al, 2015. River chloride trends in snow-affected urban watersheds...<br><br>Dugan, et al... 2017. Salting our freshwater lakes...<br><br>Kaushal et al. 2005. Increased salinization of fresh water in the northeastern United States...<br><br>Novotny et al 2009. Increase of urban lake salinity by road deicing salt...<br><br>Rusydi, 2018. Correlation between conductivity and total dissolved solids in various types of water... | Yes, we have expanded this part of the discussion, and these are great studies to cite. Although we already had references to Rusydi (2018), and Kaushal et al (2005) , we have added the other suggested references into the revised MS and expanded upon the Rusydi paper in the Methods here: "***This instrument reported "total dissolved solutes," which we converted to conductivity by dividing by 0.71, following manufacturer guidelines which are based on the physical principle that dissolved ions in water facilitate the flow of electrical current through water [36].*** " In the Discussion 5th paragraph, the other references are incorporated here: "***Chloride ions dissolved from road salt have been linked to higher conductivity in freshwater lakes and rivers in many settings, with winter pulses in chloride concentrations coinciding with the seasonal application of road salt [59–62]*** " and 9th paragraph here: "***, and we expect these numbers to grow in the future with the anticipated continued salinization of freshwater systems [60]*** " |
| ed-7 | The current analysis does not distinguish among salt application regimes (e.g., state vs. municipal roads, plow routes, salt/sand mixes) nor incorporate traffic volume or maintenance policies. Yet the discussion implies that actionable decision-making can be derived directly from the results. Add a cautionary note that policy application should be context-specific and that further work is needed to refine local salt management guidelines. | Added new sentences towards end of discussion: "***According to Massachussetts GIS data, 65% of the nearest roads to our wetlands are owned by local towns or municipalities, 9% are owned by the state Department of Transportation, 1% are owned by state conservation or recreation agencies and 25% are "unaccepted" roads that are likely privately managed. Thus, road salt application strategies are largely in the hands of local decision-makers, who may also be the ones best positioned to balance local conservation and road safety concerns. Although we have estimated the size of the mean potential salt effect zone across our entire region, this is arrived at by averaging across substantial variation. Each wetland has its own idiosyncratic relationship to the surrounding environment and thus conservation decisions for any particular site should incoprorate the specific local context. More nuanced management guidance requires further work on how the flow of road salt through the environment is controlled.*** " Also see new sentence earlier in the discussion: "***For example, one wetland that occurred only 16 m from a road had a very low conductivity of 20 µS/cm – this road occurs in a state forest where the managers close that section of road in winter rather than applying deicing salt.*** " |

| # | Comment | Response |
|---|---|---|
| ed-8 | The results state that egg masses were more numerous in high-conductivity ponds for Jefferson salamanders. This finding runs counter to the general negative relationship seen in other species and may suggest either resistance or confounding (e.g., these species breeding in less affected seasons or in hydroperiod-different pools). Clarify that data were insufficient for modeling and caution against interpreting raw egg mass counts as indicating preference or tolerance. | See new sentences in discussion "***The correlations between conductivity and amphibian populations should be interpreted cautiously. In particular, the positive association between conductivy and Jefferson salamander raw egg mass counts was based on too few points for full statistical analysis and should not be taken as indicating tolerance or preference.*** " |
| R2-1 | However, in the revised manuscript there is a need to more completely describe the models and check model fit. | In this new revision, we have provided a more robust analysis of model fit, see new sentences in Methods: "***We tested the goodness of fit of occupancy models using parametric bootstrapping with the unmarked::parboot() function[48]....We evaluated overdispersion in models with zero-inflated poisson and negative binomial error distributions using the chat() function in the nmixgof R package[50] and ultimately selected the negative binomial model to account for the clumpy distribution of egg masses. Standard errors for N-mixture models were adjusted by multiplying by √ĉ prior to reporting and calculating p-values and goodness of fit was checked with parametric bootstrapping using parboot()*** " and in Results: "***In the occupancy model for spotted salamanders there was no evidence for lack of fit in bootstrap simulations (p=0.3)... The overall occupancy model did not show strong evidence of lack of fit (p = 0.19) for wood frogs....For spotted salamanders, the N-mixture model... showed signs of moderate overdispersion within an acceptable range for ecological data (ĉ = 1.8) [52], and showed no strong evidence for lack of fit (p=0.24)..... For wood frogs,... showed no strong evidence of lack of fit (p=0.19) and had moderate but acceptable*** |
| R2-2 | For example, on line 224 the authors report a significant effect of conductivity on detection. It is unclear why conductivity would be included in the detection model. | This comment has prompted us to take a closer look at including conductivity in detection, and we discovered that it was causing instabliity in one of the models, and so has been removed. To fully reflect our thinking, see new sentence in the methods: "***We also examined occupancy models that included conductivity in the detection model to account for any potential differences in detectability related to water clarity resulting from underlying water quality,*** " and see detailed findings about inclusion of this term in the final models in each of the relevant paragraphs for each species and model in the Results sections |

| # | Comment | Response |
|---|---|---|
| R2-3 | The reader needs to know what variables were included in the detection and occupancy models specifically, and how models with different covariates included were compared. | All variables in both the detection and occupancy models are described in the methods section, under "Species Response:" "***We included as occupancy covariates distance from road, elevation, perimeter length, and forest cover, with conductivity, day of year, and canopy cover as detection covariates...To investigate potential effects of conductivity on abundance of the spotted salamanders and wood frogs, we then used the same set of predictor variables in the same occupancy framework with an N-mixture model [49] with egg mass count as the response and a 150 m radius buffer for forest canopy cover. We also used AIC to examine whether a quadratic term of conductivity would improve the abundance models to account for a unimodal response.*** " To clarify, we also added this new sentence in this revision: "***Otherwise, all detection and state variables remained the same between N-mixture and occupancy models.*** " Additionally, all variables used can be seen in the S1 Tables and in the R scripts. Please let us know if there is more we may do to clarify. |
| R2-4 | How do the authors know the data was not overdispersed? | See response to comment R2-1 above on how we checked for over-dispersion. Based on the reviewer's suggestions, we have also now adjusted standard errors and p-values to account for overdispersion, see Methods: "***Standard errors for N-mixture models were adjusted by multiplying by $\sqrt{\hat{c}}$ prior to reporting and prior to calculating p-values*** " as now noted in results with the phrase "***after correcting for overdispersion*** " to put p-values in context |
| R2-5 | Independent variables are usually scaled (observation minus mean divided by SD) to remove the effects of differences in scale among independent variables. | Originally, we scaled the independent variables using a different method, but the reviewer's suggestion is correct and all models have now been re-run with the more standard approach. See new sentence in methods: "***All independent variables were scaled by subtracting by the mean and dividing by the standard deviation prior to modeling.*** " |
| R2-6 | Where time of day and day of year included in detection models? | Time of day was not included in the models, but day of year is, see original text "***We included as occupancy covariates conductivity, distance from road, elevation, perimeter length, and forest cover, with conductivity, day of year, and canopy cover as detection*** [illegible] |
| R2-7 | I think some of this information is in the "S1 Tables" referenced on line 221 and described as Supporting Information later in the manuscript. This table is critical to assessing the manuscript but was not included with the submission material. | We have now moved all supplemental tables to the main text for ease of reading. And, yes, this information was in the S1 Tables, which were included in the appendix linked to in the revision ("S1_Tables.docx"), for some reason it seems the reviewer was unable to access this file. For the remainder of our supplemental files, if there is a better way that the journal would like for us to provide supplemental information for review, please let us know. |

| # | Comment | Response |
| --- | --- | --- |
| R2-8 | I am still concerned about the way the authors estimate the effect zone and describe it as absolute. Assuming every wetland within the estimated effect distance is affected, as the authors calculation of the percent of wetlands affected does, is not supported by the data. In fact, many wetlands well inside the estimated impact zone have conductivities below 20 and most wetlands with low conductivities are inside the estimated impact zone (Figure 2 of the current draft). What might be most troubling here is those wetlands that have higher conductivities but are well outside the estimated impact zone. I am not sure the analyses need to be redone, but I do think the authors have to acknowledge some how that estimating a specific distance can be misleading. They do this in the discussion with smaller wetlands within the effect zone. However, this does not deal with wetlands outside the effect zone that might be affected by road salt. Based on Figure 2, there are quite a few wetlands that have conductivities that align with the average within the effect zone. Is it possible that the authors approach greatly underestimates the potential impact zone? | We added new paragraphs addressing these concerns in the discussion, happy to revise futher as needed: See new sentences: "***Here, we have modeled mean conductivity across hundreds of sites, whereas the chemistry of any particular wetland is controlled by myriad complex factors related to human decision making and environmental setting. For example, one wetland that occurred only 16 m from a road had a very low conductivity of 20 µS/cm – this road occurs in a state forest where the managers close that section of road in winter rather than applying deicing salt. Another road that occurs 89 m from a road but had a conductivity reading of only 3 µS/cm appears to be a dirt road on satellite imagery, and thus is unlikely to be treated with salt. The patterns observed at these two sites are consistent with our overall conclusion that elevated wetland conductivity is driven by road salt application... although most of our sites are mapped as forested at the point locations, runoff from adjacent lands such as nearby farms, developments, or natural features may also contribute elevated salinity to our focal wetlands. For example, two wetlands in our study that lie 1060 m and 1260 m from the nearest road measured conductivities of 130 µS/cm and 113 µS/cm, respectively. Relative to other wetlands this far from roads, these readings appear high, however, both of these ponds are positioned immediately downslope from blocks of basalt bedrock which likely releases high levels of cations, elevating conductivity of those wetlands [60]. Similarly, two wetlands 760 m and 1060 m from the nearest road also have relatively high conductivities of 196 µS/cm and 330 µS/cm, respectively. Both of these wetlands appear to be hydrologically connected to much larger swamps that may transport ions from a variety of sources on the landscape of both non-human and human origin.*** " |
| R2-9 | Lines 129 - 131: log transformation of conductivity is done to more closely approximate a normal distribution of the dependent variable. Log transformation of independent variables is done to accommodate nonlinear responses. | Revised sentence as suggested to read: "Prior to regression analyses, to obtain a more normal distribution in the response variable, we log-transformed water conductivity and to obtain a more linear relationship between response and predictor, we log-transformed the distance to nearest road. " |
| R2-10 | Line 143: …fitting the initial model with all wetlands, we iteratively fit the model… | Added the missing "fit" to correct syntax. |
| R2-11 | Lines 146-147: We then defined the end of the potential road effect zone as the distance at which the estimated… To the point that wetlands inside the zone did not always have elevated conductivities, this is better termed a potential road effect zone. | Added "potential" as suggested by the reviewer. |
| R2-12 | Line 165: delete period in the middle of the sentence. | corrected, thank you. |

| # | Comment | Response |
|---|---|---|
| R2-13 | Line 202: …our estimated potential salt effect zone… | Added "potential" as suggested by the reviewer. |
| R2-14 | Lines 244-246: Or smaller wetlands have smaller watershed sizes that are less likely to intersect the road the smaller the wetland is. | Yes, we added the reviewer's idea to the subsequent sentence that suggests that smaller wetlands may have fewer surface water inputs, which now reads: "***These smaller wetlands may also have smaller watersheds intersecting fewer roads and  fewer connections to flowing surface and ground water that may transport salt to the wetlands.*** " |
| R2-15 | What is occurrence in figure 4? | Changed y-axis to "Number of Occurrences" to clarify that the bar heights represent the number of ponds at which we saw the given species at a site with the given conductivity. |
