# Supplementary material for: The reach of road salt into vernal pools and the response of amphibians
Source: PLoS One. 2025 Oct 23;20(10):e0329680. doi: 10.1371/journal.pone.0329680 (PMC12548906; doi:10.1371/journal.pone.0329680)
Supplement: Peer review history

# Peer review: Decision Letter 0

**DOI:** 10.1371/journal.pone.0329680.r001

**Authors:** Petr Heneberg

18 Feb 2025

Dear Dr. Charney,

Thank you for submitting your manuscript to PLOS ONE. After careful consideration, we feel that it has merit but does not fully meet PLOS ONE’s publication criteria as it currently stands. Therefore, we invite you to submit a revised version of the manuscript that addresses the points raised during the review process.

<

Please submit your revised manuscript by Apr 04 2025 11:59PM. If you will need more time than this to complete your revisions, please reply to this message or contact the journal office at plosone@plos.org . When you're ready to submit your revision, log on to https://www.editorialmanager.com/pone/ and select the 'Submissions Needing Revision' folder to locate your manuscript file.

- A rebuttal letter that responds to each point raised by the academic editor and reviewer(s). You should upload this letter as a separate file labeled 'Response to Reviewers'.
- A marked-up copy of your manuscript that highlights changes made to the original version. You should upload this as a separate file labeled 'Revised Manuscript with Track Changes'.
- An unmarked version of your revised paper without tracked changes. You should upload this as a separate file labeled 'Manuscript'.

If you would like to make changes to your financial disclosure, please include your updated statement in your cover letter. Guidelines for resubmitting your figure files are available below the reviewer comments at the end of this letter.

If applicable, we recommend that you deposit your laboratory protocols in protocols.io to enhance the reproducibility of your results. Protocols.io assigns your protocol its own identifier (DOI) so that it can be cited independently in the future. For instructions see: https://journals.plos.org/plosone/s/submission-guidelines#loc-laboratory-protocols . Additionally, PLOS ONE offers an option for publishing peer-reviewed Lab Protocol articles, which describe protocols hosted on protocols.io. Read more information on sharing protocols at https://plos.org/protocols?utm_medium=editorial-email&utm_source=authorletters&utm_campaign=protocols .

We look forward to receiving your revised manuscript.

Kind regards,

Petr Heneberg

Academic Editor

PLOS ONE

Journal requirements:

When submitting your revision, we need you to address these additional requirements.

1. Please ensure that your manuscript meets PLOS ONE's style requirements, including those for file naming. The PLOS ONE style templates can be found at

https://journals.plos.org/plosone/s/file?id=wjVg/PLOSOne_formatting_sample_main_body.pdf and

https://journals.plos.org/plosone/s/file?id=ba62/PLOSOne_formatting_sample_title_authors_affiliations.pdf

2. In your Methods section, please provide additional information regarding the permits you obtained for the work. Please ensure you have included the full name of the authority that approved the field site access and, if no permits were required, a brief statement explaining why.

3. Thank you for stating the following financial disclosure:  [The Natural Heritage & Endangered Species Program of the Massachusetts Division of Fisheries and Wildlife; the National Science Foundation Graduate Research Fellowship Program; The Amherst College Lloyd I Rosenblum Memorial Fellowship Program; McIntire-Stennis funding, project award no. ME0-42312, from the U.S. Department of Agriculture’s National Institute of Food and Agriculture.].  Please state what role the funders took in the study.  If the funders had no role, please state: "The funders had no role in study design, data collection and analysis, decision to publish, or preparation of the manuscript." If this statement is not correct you must amend it as needed. Please include this amended Role of Funder statement in your cover letter; we will change the online submission form on your behalf.

4. When completing the data availability statement of the submission form, you indicated that you will make your data available on acceptance. We strongly recommend all authors decide on a data sharing plan before acceptance, as the process can be lengthy and hold up publication timelines. Please note that, though access restrictions are acceptable now, your entire data will need to be made freely accessible if your manuscript is accepted for publication. This policy applies to all data except where public deposition would breach compliance with the protocol approved by your research ethics board. If you are unable to adhere to our open data policy, please kindly revise your statement to explain your reasoning and we will seek the editor's input on an exemption. Please be assured that, once you have provided your new statement, the assessment of your exemption will not hold up the peer review process. 

5. Please include a caption for figure 4.

Additional Editor Comments (if provided):

[Note: HTML markup is below. Please do not edit.]

Reviewers' comments:

Reviewer's Responses to Questions

**Comments to the Author**

1. Is the manuscript technically sound, and do the data support the conclusions?

Reviewer #1: Partly

Reviewer #2: No

**********

2. Has the statistical analysis been performed appropriately and rigorously?

Reviewer #1: No

Reviewer #2: No

**********

3. Have the authors made all data underlying the findings in their manuscript fully available?

The PLOS Data policy

Reviewer #1: No

Reviewer #2: Yes

**********

4. Is the manuscript presented in an intelligible fashion and written in standard English?

Reviewer #1: Yes

Reviewer #2: No

**********

Reviewer #1: Dear Authors. Presentet research focuse on important topic of freshwater salinization, in which there are still many knowledge gaps. Your manuscript give some new knowledge, but before publication it needs some improvement listed below.

Main comments

•First of all, I suggest change title – there are two main topics in manuscript – salinization of wetland at difference length from road and its impact on amphibian population – title should include both topics. Off course you mention also about shrimps, if title would not be long you can add it too.

•Secondly, you results presented half to half information about salinity vs road distant, and salinity vs amphibians – but you only present on figures first one. You would be very valuable to present some more figures/tables with salinity vs amphibians analyses, including some statistical. You make huge work to investigate such a number of wetlands – it should be a lot data base to make some statistic analyses.

•You collected data almost 15 years ago. It no bad, because they are still give new knowledge, but you discussion part (or rather the entire manuscript) compare you research with newer publications (only 7 from 45 are published after 2015). There are many new publication and some older publication, about relation of salinization and amphibians, like for example: https://doi.org/10.1890/ES14-00534.1; https://doi.org/10.1007/s00442-022-05243-3; https://doi.org/10.1002/ece3.11069; https://doi.org/10.1643/h2020070; https://doi.org/10.1016/j.envpol.2016.11.060; https://doi.org/10.1080/02705060.2009.9664301

•In Material and Methods you give information about number of days with max temperature below 0 °C, but road salt is also use when max is above 0 °C, and for night temperature falling below this value. Please add this information, which is very important in relation to road salt application

•There is no information about wetland base characteristic – area – you are using GIS, so it should not be a problem to add this.

•Wetland area is important in relation with TDS measurement – if wetland are small, you can measure in one place, but what if they are large? It important, because as you wrote about different hydrological connection of wetlands. If all were similar you can measure for example at inflow part. But for now this information is missed in manuscript.

•Related to above, similar question about amphibian and shrimp investigation – on which part of wetland you analyse them.

•Finally, you collect a lot of data, but you don’t meet Plos One standards -

“The PLOS Data policy requires authors to make all data underlying the findings described in their manuscript fully available without restriction, with rare exception (please refer to the Data Availability Statement in the manuscript PDF file). The data should be provided as part of the manuscript or its supporting information, or deposited to a public repository. For example, in addition to summary statistics, the data points behind means, medians and variance measures should be available. If there are restrictions on publicly sharing data—e.g. participant privacy or use of data from a third party—those must be specified.”

Reviewer #2: Overall, I think the goals of this paper are worthwhile and that the authors have data that could possibly be used to address the questions presented. However, I have a number of concerns with the current manuscript that make it difficult to recommend publication. The authors may be able to address these concerns in a way that leads to a recommendation to publish, but because these concerns include several areas of clarification concerning analyses and would require reanalysis in several areas, it is impossible to know how addressing my concerns might affect any conclusions that are drawn. Below I outline my major concerns and well as some more minor correction.

1) It is well-recognized that detection of amphibian use of wetlands is subject to false negatives (lack of detection when the wetland is actually used). The authors seem to collect data that might let them estimate detection probabilities as 51 wetlands were visited two years in a row. It is unclear why the authors made no attempt to include detection errors in their modeling efforts. Concerns around detection error are particularly concerning for explosive breeders such as wood frogs. On a related note, the authors should also consider date of visit in their analyses for both conductivity and amphibian occurrence as detection probabilities and road salt effects are expected to diminish later in spring and summer.

2) Wetland hydroperiod has been well established as a primary controller of community structure in vernal pool and other depression wetland systems. It seems to draw any conclusion concerning the effects of road salts on amphibian use of wetland, the authors would need to include some measures of hydroperiod as a covariate in any of their analyses.

3) Lines 106-107: I am not familiar with this approach. The authors convert TDS to conductivity by simply dividing by 0.71. There is no information presented on this relationship or its relationship to ions associated with road salts. With no information on the relationship between Real Salt associated ions and TDS it is impossible to know that TDS is measuring road salt impacts.

4) It is not clear why the authors made no attempt to include detection probabilities in their analyses of amphibian occurrence. The amphibians that were included in the study are seasonal and explosive breeders, making false negatives a real possibility. The authors did visit 51 wetlands twice, so they have the data to estimate detection probabilities and correct estimates of occurrence accordingly. However, they assume these data are 100% accurate and analyze transition probabilities. This assumption makes it hard to draw any conclusions concerning impacts distances. On another but related note, the authors should use day of year in their analyses of both conductivity and amphibian occurrence as they are both expected to decline later in spring and summer.

5) Lines 133-134: The authors note they transformed variables to assure normality. They do not appear to have checked for normality of conductivity in the regression models. Moreover, independent variables such as distance from roads are transformed to achieve linearity. Therefore, most important here is the effects of this transformation on their estimate of distance effects. How would their results change if they did not log-transform distance from roads?

6) There is confusion on model description throughout the manuscript. On lines 138-140 and again on lines 149-151 the authors descript a series of independent variables, including distance from roads, as random effects and region as a fixed effect. This should be the other way around with region as the random effect and the other variables as fixed (because we expect for example that TDS will decline with distance from roads). Moreover, in table 1 the fixed and random effects appear to be describe correctly, but the legend indicates a logistic regression when this is clear results from a linear regression.

7) Lines 158-159: egg mass counts would be more appropriately analyzed with a poisson error term.

8) Lines 184-185: This seems to be too simple an approach. I assume this is simply a spatial analysis conducted by buffering roads. There are wetlands well inside of the estimated effect zone that have the lowest conductivities while there are wetlands well outside of the effect zone that have conductivities above 200 microS/cm. What is really interesting here are these outliers. What cause them-could it be road type or traffic volume? We know that road salt effects decline with distance from roads. The next step should be figuring out what causes variation in effect distances and how they relate to things we can manage. I think this paper could be much more interests if information on road characteristics were included in some way.

**********

what does this mean? ). If published, this will include your full peer review and any attached files.

If you choose “no”, your identity will remain anonymous but your review may still be made public.

**Do you want your identity to be public for this peer review?** For information about this choice, including consent withdrawal, please see our Privacy Policy

Reviewer #1: **Yes: ** Sebastian Szklarek

Reviewer #2: No

**********

[NOTE: If reviewer comments were submitted as an attachment file, they will be attached to this email and accessible via the submission site. Please log into your account, locate the manuscript record, and check for the action link "View Attachments". If this link does not appear, there are no attachment files.]

While revising your submission, please upload your figure files to the Preflight Analysis and Conversion Engine (PACE) digital diagnostic tool, https://pacev2.apexcovantage.com/ . PACE helps ensure that figures meet PLOS requirements. To use PACE, you must first register as a user. Registration is free. Then, login and navigate to the UPLOAD tab, where you will find detailed instructions on how to use the tool. If you encounter any issues or have any questions when using PACE, please email PLOS at figures@plos.org . Please note that Supporting Information files do not need this step.

# Author comment: Author response to Decision Letter 1

**DOI:** 10.1371/journal.pone.0329680.r002

5 Jun 2025

Please see responses upload as instructed by PLOSOne staff

Attachment Submitted filename: line-by-line_revision1_V1.pdf

# Peer review: Decision Letter 1

**DOI:** 10.1371/journal.pone.0329680.r003

**Authors:** Petr Heneberg

27 Jun 2025

Dear Dr. Charney,

Thank you for submitting your manuscript to PLOS ONE. After careful consideration, we feel that it has merit but does not fully meet PLOS ONE’s publication criteria as it currently stands. Therefore, we invite you to submit a revised version of the manuscript that addresses the points raised during the review process.

Please submit your revised manuscript by Aug 11 2025 11:59PM. If you will need more time than this to complete your revisions, please reply to this message or contact the journal office at plosone@plos.org . When you're ready to submit your revision, log on to https://www.editorialmanager.com/pone/ and select the 'Submissions Needing Revision' folder to locate your manuscript file.

- A rebuttal letter that responds to each point raised by the academic editor and reviewer(s). You should upload this letter as a separate file labeled 'Response to Reviewers'.
- A marked-up copy of your manuscript that highlights changes made to the original version. You should upload this as a separate file labeled 'Revised Manuscript with Track Changes'.
- An unmarked version of your revised paper without tracked changes. You should upload this as a separate file labeled 'Manuscript'.

If applicable, we recommend that you deposit your laboratory protocols in protocols.io to enhance the reproducibility of your results. Protocols.io assigns your protocol its own identifier (DOI) so that it can be cited independently in the future. For instructions see: https://journals.plos.org/plosone/s/submission-guidelines#loc-laboratory-protocols . Additionally, PLOS ONE offers an option for publishing peer-reviewed Lab Protocol articles, which describe protocols hosted on protocols.io. Read more information on sharing protocols at https://plos.org/protocols?utm_medium=editorial-email&utm_source=authorletters&utm_campaign=protocols .

We look forward to receiving your revised manuscript.

Kind regards,

Petr Heneberg

Academic Editor

PLOS ONE

Journal Requirements:

Please review your reference list to ensure that it is complete and correct. If you have cited papers that have been retracted, please include the rationale for doing so in the manuscript text, or remove these references and replace them with relevant current references. Any changes to the reference list should be mentioned in the rebuttal letter that accompanies your revised manuscript. If you need to cite a retracted article, indicate the article’s retracted status in the References list and also include a citation and full reference for the retraction notice.

Additional Editor Comments (if provided):

The abstract summarizes the main findings clearly and concisely. However, the abstract should clarify that the amphibian models focused only on species with sufficient data (e.g., spotted salamanders and wood frogs), and that occupancy modeling was not extended to all taxa mentioned.

Potentially controversial claim – salt as sole conductivity driver. The manuscript implies that elevated conductivity is predominantly due to road salt. While plausible, this assumption could be overstated. The authors acknowledge other potential sources (fertilizers, metals, etc.) in the discussion but minimize them with a reference to parsimony and prior literature. Yet, no ion-specific measurements (e.g., chloride, sodium) are presented. Including some ion analysis, or at least referencing more detailed studies linking specific ion concentrations to road salt, would strengthen this assertion.

Justify the 167–251 m threshold as ecologically meaningful or offer a sensitivity analysis using alternative definitions (e.g., specific conductivity thresholds, modeled change points, or species response breakpoints).

Consider including NLCD land cover type as a categorical predictor or discussing in greater detail how omitted variables might bias estimates.

The wood frog occupancy model did not find a significant effect of conductivity, yet strong inferences are made in the discussion based on abundance models. These interpretations could mislead readers into assuming stronger evidence of effect than warranted. Tone down conclusions related to wood frog occupancy and clarify that the effect was detected only in abundance and with uncertainty in peak location (68 μS/cm).

Some additional key recent works could enhance the discussion, especially regarding ion-specific toxicity and landscape modeling of salt impacts.

The current analysis does not distinguish among salt application regimes (e.g., state vs. municipal roads, plow routes, salt/sand mixes) nor incorporate traffic volume or maintenance policies. Yet the discussion implies that actionable decision-making can be derived directly from the results. Add a cautionary note that policy application should be context-specific and that further work is needed to refine local salt management guidelines.

The results state that egg masses were more numerous in high-conductivity ponds for Jefferson salamanders. This finding runs counter to the general negative relationship seen in other species and may suggest either resistance or confounding (e.g., these species breeding in less affected seasons or in hydroperiod-different pools). Clarify that data were insufficient for modeling and caution against interpreting raw egg mass counts as indicating preference or tolerance.

Clarify in the data availability statement which fields or datasets are restricted and under what terms.

References to be reflected:

Corsi, S.R., De Cicco, L.A., Lutz, M.A. and Hirsch, R.M., 2015. River chloride trends in snow‐affected urban watersheds: Increasing concentrations outpace urban growth rate and are common among all seasons. Science of the Total Environment, 508, pp.488–497. https://doi.org/10.1016/j.scitotenv.2014.12.012

Dugan, H.A., Bartlett, S.L., Burke, S.M., Doubek, J.P., Krivak‐Tetley, F.E., Skaff, N.K., Summers, J.C., Farrell, K.J., McCullough, I.M., Morales‐Williams, A.M. and Roberts, D.C., 2017. Salting our freshwater lakes. Proceedings of the National Academy of Sciences, 114(17), pp.4453–4458. https://doi.org/10.1073/pnas.1620211114

Kaushal, S.S., Groffman, P.M., Likens, G.E., Belt, K.T., Stack, W.P., Kelly, V.R., Band, L.E. and Fisher, G.T., 2005. Increased salinization of fresh water in the northeastern United States. Proceedings of the National Academy of Sciences, 102(38), pp.13517–13520. https://doi.org/10.1073/pnas.0506414102

Novotny, E.V., Murphy, D. and Stefan, H.G., 2009. Increase of urban lake salinity by road deicing salt. Science of the Total Environment, 406(1–2), pp.131–144. https://doi.org/10.1016/j.scitotenv.2008.07.037

Rusydi, A.F., 2018. Correlation between conductivity and total dissolved solids in various types of water: A review. IOP Conference Series: Earth and Environmental Science, 118(1), p.012019. https://doi.org/10.1088/1755-1315/118/1/012019

[Note: HTML markup is below. Please do not edit.]

Reviewers' comments:

Reviewer's Responses to Questions

**Comments to the Author**

Reviewer #1: All comments have been addressed

Reviewer #2: (No Response)

**********

2. Is the manuscript technically sound, and do the data support the conclusions?

Reviewer #1: Yes

Reviewer #2: Partly

**********

3. Has the statistical analysis been performed appropriately and rigorously?

Reviewer #1: Yes

Reviewer #2: I Don't Know

**********

4. Have the authors made all data underlying the findings in their manuscript fully available?

The PLOS Data policy

Reviewer #1: Yes

Reviewer #2: No

**********

5. Is the manuscript presented in an intelligible fashion and written in standard English?

Reviewer #1: Yes

Reviewer #2: Yes

**********

Reviewer #1: Thank you for consider all comments. I don`t have more. Manuscript might be now published in the journal.

Reviewer #2: I want to thank the authors for incorporation of the occupancy and in n-mixture models. Both model types appear to be used appropriately in relationship to response variable type. However, in the revised manuscript there is a need to more completely describe the models and check model fit. For example, on line 224 the authors report a significant effect of conductivity on detection. It is unclear why conductivity would be included in the detection model. The reader needs to know what variables were included in the detection and occupancy models specifically, and how models with different covariates included were compared. How do the authors know the data was not overdispersed? Independent variables are usually scaled (observation minus mean divided by SD) to remove the effects of differences in scale among independent variables. Where time of day and day of year included in detection models? I think some of this information is in the “S1 Tables” referenced on line 221 and described as Supporting Information later in the manuscript. This table is critical to assessing the manuscript but was not included with the submission material.

I am still concerned about the way the authors estimate the effect zone and describe it as absolute. Assuming every wetland within the estimated effect distance is affected, as the authors calculation of the percent of wetlands affected does, is not supported by the data. In fact, many wetlands well inside the estimated impact zone have conductivities below 20 and most wetlands with low conductivities are inside the estimated impact zone (Figure 2 of the current draft). What might be most troubling here is those wetlands that have higher conductivities but are well outside the estimated impact zone. I am not sure the analyses need to be redone, but I do think the authors have to acknowledge some how that estimating a specific distance can be misleading. They do this in the discussion with smaller wetlands within the effect zone. However, this does not deal with wetlands outside the effect zone that might be affected by road salt. Based on Figure 2, there are quite a few wetlands that have conductivities that align with the average within the effect zone. Is it possible that the authors approach greatly underestimates the potential impact zone?

Some more minor comments:

Lines 129 - 131: log transformation of conductivity is done to more closely approximate a normal distribution of the dependent variable. Log transformation of independent variables is done to accommodate nonlinear responses.

Line 143: …fitting the initial model with all wetlands, we iteratively fit the model…

Lines 146-147: We then defined the end of the potential road effect zone as the distance at which the estimated… To the point that wetlands inside the zone did not always have elevated conductivities, this is better termed a potential road effect zone.

Line 165: delete period in the middle of the sentence.

Line 202: …our estimated potential salt effect zone...

Lines 244-246: Or smaller wetlands have smaller watershed sizes that are less likely to intersect the road the smaller the wetland is.

What is occurrence in figure 4?

**********

what does this mean? ). If published, this will include your full peer review and any attached files.

If you choose “no”, your identity will remain anonymous but your review may still be made public.

**Do you want your identity to be public for this peer review?** For information about this choice, including consent withdrawal, please see our Privacy Policy

Reviewer #1: **Yes: ** Sebastian Szklarek

Reviewer #2: No

**********

[NOTE: If reviewer comments were submitted as an attachment file, they will be attached to this email and accessible via the submission site. Please log into your account, locate the manuscript record, and check for the action link "View Attachments". If this link does not appear, there are no attachment files.]

While revising your submission, please upload your figure files to the Preflight Analysis and Conversion Engine (PACE) digital diagnostic tool, https://pacev2.apexcovantage.com/ . PACE helps ensure that figures meet PLOS requirements. To use PACE, you must first register as a user. Registration is free. Then, login and navigate to the UPLOAD tab, where you will find detailed instructions on how to use the tool. If you encounter any issues or have any questions when using PACE, please email PLOS at figures@plos.org

# Author comment: Author response to Decision Letter 2

**DOI:** 10.1371/journal.pone.0329680.r004

17 Jul 2025

Please see separate line-by-line responses in uploaded Response to Reviewers file

Attachment Submitted filename: vpSalt_SecondReviewerComments_v5.pdf

# Editor report: Decision Letter 2

**DOI:** 10.1371/journal.pone.0329680.r005

**Authors:** Petr Heneberg

21 Jul 2025

The reach of road salt into vernal pools and the response of amphibians

PONE-D-24-59281R2

Dear Dr. Charney,

We’re pleased to inform you that your manuscript has been judged scientifically suitable for publication and will be formally accepted for publication once it meets all outstanding technical requirements.

Within one week, you’ll receive an e-mail detailing the required amendments. When these have been addressed, you’ll receive a formal acceptance letter and your manuscript will be scheduled for publication.

An invoice will be generated when your article is formally accepted. Please note, if your institution has a publishing partnership with PLOS and your article meets the relevant criteria, all or part of your publication costs will be covered. Please make sure your user information is up-to-date by logging into Editorial Manager at Editorial Manager®  and clicking the ‘Update My Information' link at the top of the page. If you have any questions relating to publication charges, please contact our Author Billing department directly at authorbilling@plos.org.

If your institution or institutions have a press office, please notify them about your upcoming paper to help maximize its impact. If they’ll be preparing press materials, please inform our press team as soon as possible -- no later than 48 hours after receiving the formal acceptance. Your manuscript will remain under strict press embargo until 2 pm Eastern Time on the date of publication. For more information, please contact onepress@plos.org.

Kind regards,

Petr Heneberg

Academic Editor

PLOS ONE

Additional Editor Comments (optional):

Reviewers' comments:

# Editor report: Acceptance letter

**DOI:** 10.1371/journal.pone.0329680.r006

**Authors:** Petr Heneberg

PONE-D-24-59281R2

PLOS ONE

Dear Dr. Charney,

I'm pleased to inform you that your manuscript has been deemed suitable for publication in PLOS ONE. Congratulations! Your manuscript is now being handed over to our production team.

At this stage, our production department will prepare your paper for publication. This includes ensuring the following:

* All references, tables, and figures are properly cited

* All relevant supporting information is included in the manuscript submission,

* There are no issues that prevent the paper from being properly typeset

You will receive further instructions from the production team, including instructions on how to review your proof when it is ready. Please keep in mind that we are working through a large volume of accepted articles, so please give us a few days to review your paper and let you know the next and final steps.

Lastly, if your institution or institutions have a press office, please let them know about your upcoming paper now to help maximize its impact. If they'll be preparing press materials, please inform our press team within the next 48 hours. Your manuscript will remain under strict press embargo until 2 pm Eastern Time on the date of publication. For more information, please contact onepress@plos.org.

You will receive an invoice from PLOS for your publication fee after your manuscript has reached the completed accept phase. If you receive an email requesting payment before acceptance or for any other service, this may be a phishing scheme. Learn how to identify phishing emails and protect your accounts at https://explore.plos.org/phishing.

If we can help with anything else, please email us at customercare@plos.org.

Thank you for submitting your work to PLOS ONE and supporting open access.

Kind regards,

PLOS ONE Editorial Office Staff

on behalf of

Dr. Petr Heneberg

Academic Editor

PLOS ONE
